# Conformational Analysis of Novel Benzene-1,3-Disulfonamide-Based Cycloalkynes Through X-Ray Crystallography, DFT Calculations, and NMR Spectroscopy

**DOI:** 10.3390/molecules31142462

**Published:** 2026-07-14

**Authors:** Kyosuke Kaneda, Takato Koideya, Hitomi Tsuda, Haruto Katakura, Haruhiko Fukaya, Takehiro Yamagishi

**Affiliations:** 1 Department of Medicinal Chemistry, Faculty of Pharmaceutical Sciences, Hokkaido University of Science, 7-15-4-1, Maeda Teine, Sapporo 006-8585, Hokkaido, Japanyamagishi-t@hus.ac.jp (T.Y.); 2 School of Pharmacy, Tokyo University of Pharmacy and Life Sciences, 1432-1 Horinouchi, Hachioji 192-0392, Tokyo, Japan; fukayah@toyaku.ac.jp

**Keywords:** sulfonamide, cycloalkyne, strained conformation, X-ray crystallography, nuclear magnetic resonance

## Abstract

Sulfonamides are a fundamental class of compounds with diverse pharmacological applications. Here, two benzene-1,3-disulfonamide-containing cycloalkyne compounds were designed to demonstrate the strained conformations due to the 11-membered ring. Their structures were experimentally analyzed using single-crystal X-ray crystallography and nuclear magnetic resonance (NMR) spectroscopy. The molecules exhibit flexible sulfonamide conformations together with characteristic distortions of the benzene and alkyne moieties. Comparing the compound data obtained, the proton NMR chemical shift of hydrogen at the 2-position of the benzene ring shows a correlation of a dihedral angle involving the benzene ring and sulfonamide sulfur, and the carbon NMR shift suggests an angle distortion of the alkyne. The conformations of the crystal structure and the solution state in DMSO are supported by NOESY spectra and DFT calculations. The relative chemical shift differences were quantitatively reproduced by DFT calculations. We believe this fundamental research will contribute to the design and development of sulfonamide–alkyne–benzene-based medium-sized heterocyclic molecules with detailed conformation predictions.

## 1. Introduction

**Figure 1:**
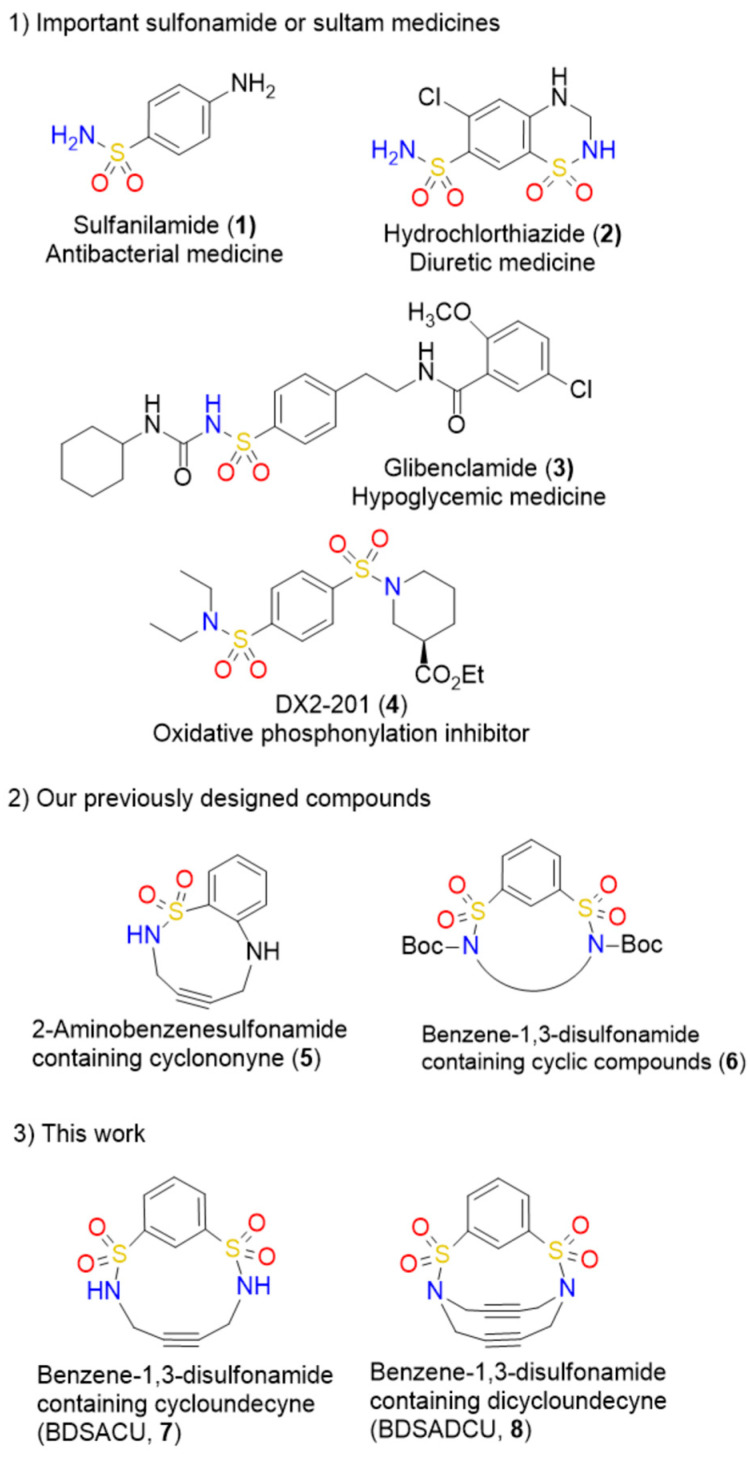
Background and concept of this research.

Sulfonamide (-SO_2_NH-) and sultam-containing medicines, which exhibit various pharmacological activities, include sulfa antibiotics (e.g., sulfanilamide, **1**), thiazide diuretics (e.g., hydrochlorothiazide, **2**) and sulfonylurea hypoglycemic drugs (e.g., glipalamide, **3**) (Figure 1) [1,2]. Recently, benzene-di-sulfonamide has gained attention as it led to the discovery of oxidative phosphorylation inhibitors such as DX2-201 (**4**) [3].

Sulfonamides, characterized by a sulfur atom bonded to two oxygen atoms and one nitrogen atom, have undergone detailed conformational studies in experimental and computational sciences. However, predicting their exact structure and conformation remains challenging [4,5,6]. This complexity arises from the structural flexibility of sulfonamides, which likely influences their diverse pharmacological activities through various interactions with functional proteins.

Since 2017, we have been investigating the design and synthesis of sultam compounds, such as 2-aminobenzenesulfonamide-containing cyclononyne (ABSACN, **5**) and elucidating their structural characteristics [7,8,9]. Continuing this research, we examined medium-sized ring sulfonamides, including benzene-1,3-disulfonamide-containing cyclic compounds (**6**) [10]. Medium-sized rings exhibit moderate conformational freedom, rendering their structural analysis particularly intriguing. In this study, we compare the crystal structures and nuclear magnetic resonance (NMR) parameters of benzene-1,3-disulfonamide-containing cycloundecyne (BDSACU, **7**) and benzene-1,3-disulfonamide-containing dicycloundecyne (BDSADCU, **8**), demonstrating the conformational flexibility involving two sulfonamide bonds, including benzene and alkyne moieties.

**Figure 2:**
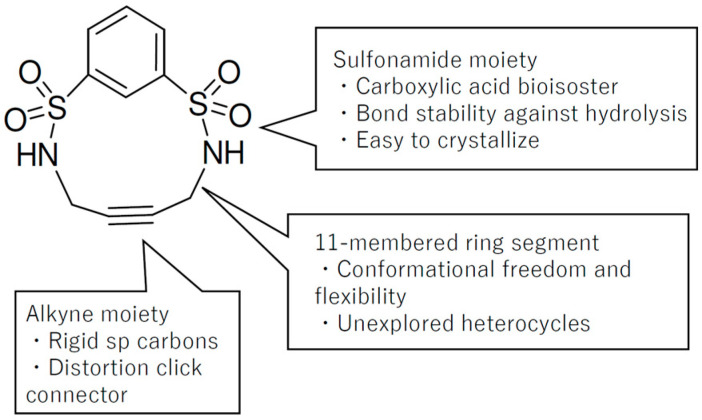
Design intention of molecule **7**.

Before describing the actual synthesis and comparing the results, we explain the molecule design of BDSACU **7**. From a medicinal chemistry perspective, sulfonamides, which are carboxylic acid bioisosteres, are inherently expected to exhibit biological activity (Figure 2) [11].

They possess bond stability in biological systems, particularly in a state that resists hydrolysis or hydrolytic stability because of resonance and three-dimensional bulkiness [12]. Experimentally, sulfonamides are relatively easy to crystallize because of their ability to function as both a hydrogen bond donor and acceptor, facilitating crystal structure analysis [13].

The 11-membered ring segment offers a degree of conformational freedom and flexibility, and their syntheses still change depending on the conformation, making them an interesting research subject in organic chemistry [14]. In addition, this structure is absent in compound databases such as Reaxys and serves to complement the chemical space of heterocyclic libraries [15].

The whole structure may be classified as a di-sulfonamide-containing hetero-meta [8] cyclophyne [16,17]. Moreover, the incorporation of an alkyne enhances the compound’s cyclic rigidity and provides reactivity, making it a promising candidate for click reaction applications [18].

Therefore, the intention behind these new 11-membered ring molecules, including sulfonamides, alkynes, and benzenes, is to expand a new chemical space. As organic molecules, they represent compounds with high potential in medicinal and structural chemistry.

## 2. Results and Discussion

Here, we show the chemical synthesis of new molecules, including a standard molecule. A detailed comparison list of these data was acquired from structural analyses such as X-ray crystallography and NMR. Furthermore, regarding the most stable conformation of the novel compound, theoretical NMR values for the DMSO solution—derived from X-ray crystal structure analysis—were determined using computational methods, thereby confirming a certain degree of equivalence between the crystalline and solution states. Additionally, as the differences in values between the compounds are consistent, we consider there to be no issues in establishing a correlation.

### 2.1. Chemical Synthesis of Standard Molecule ***12***

**Scheme 1:**
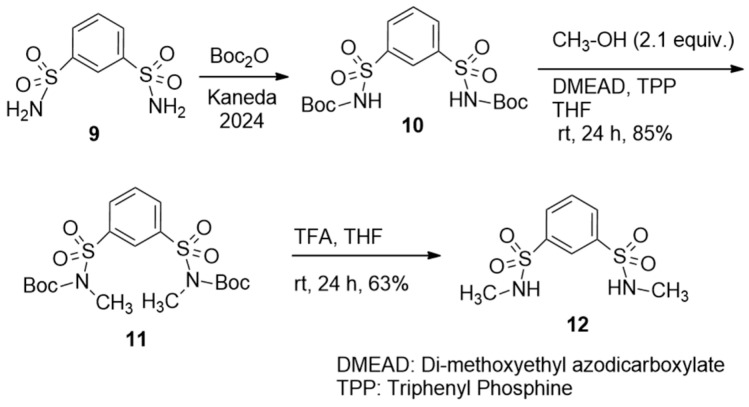
Synthesis of *N*,*N*’-dimethyl benzene-1.3-disulfonamide (**12**) [10].

We first synthesized *N*,*N*’-dimethyl-functionalized benzene-1,3-disulfonamide (**12**) as a standard compound via a double Mitsunobu alkylation reaction [10]. The detailed procedure is described in the Materials and Methods section (conversion from compound **9** to **10** in Scheme 1). We employed dimethoxyethyl azodicarboxylate (DMEAD) and triphenylphosphine (TPP) as Mitsunobu reagents, achieving a yield of 85% for intermediate compound **11** [19]. Subsequent treatment with trifluoroacetic acid (TFA) facilitated the removal of the Boc group, resulting in the desired compound **12** with a yield of 63%.

### 2.2. Chemical Synthesis of Designed Molecules ***7*** and ***8***

**Scheme 2:**
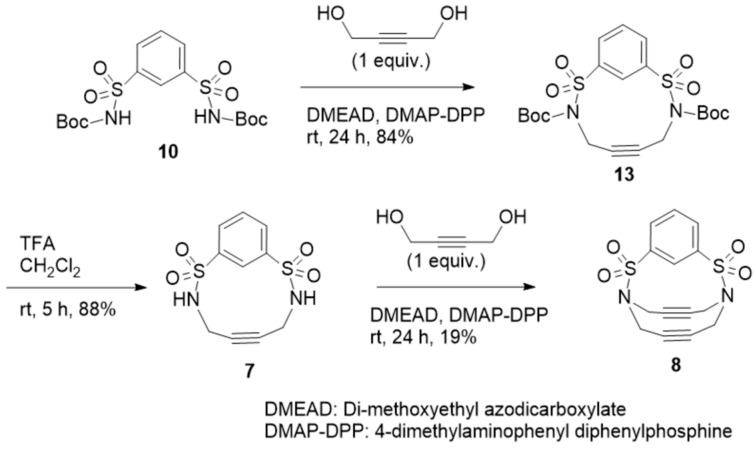
Synthesis of compounds **7** and **8**.

A double Mitsunobu cyclization reaction with but-2-yne-1,4-diol, using dimethoxyethyl azodicarboxylate (DMEAD) and 4-dimethylaminophenyl diphenylphosphine (DMAP-DPP), yielded the 11-membered ring product, *N,N’*-di-Boc-protected benzene-1,3-disulfonamide-containing cycloundecyne (**13**), with an 84% yield (Scheme 2) [20]. The removal of both Boc groups using TFA resulted in compound **7**. Remarkably, a subsequent double Mitsunobu cyclization of compound **7** with but-2-yne-1,4-diol produced another cycloundecyne ring moiety, forming compound **8** in 19% yield. The formation of bridged sulfonamides within medium-sized rings is a rare achievement in synthetic organic chemistry [21].

### 2.3. X-Ray Crystallographic Analysis of Each Molecule

**Figure 3:**
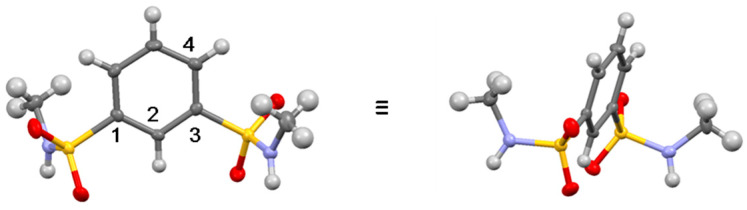
Crystal graphic of compound **12** (CCDC No. 2402057).

As shown in Figure 3, the standard compound **12** was recrystallized in AcOEt, and subsequent single-crystal X-ray crystallography provided a detailed depiction of its structure (CCDC No. 2402057). The detailed X-ray crystal structure data sees the Supplementary Materials.

**Figure 4:**
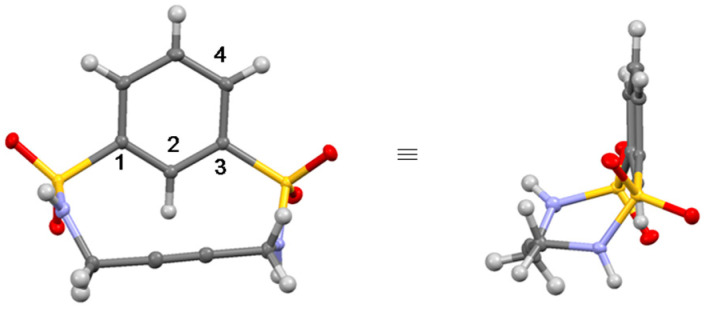
Crystal graphic of compound **7** (CCDC No. 2402073).

**Figure 5:**
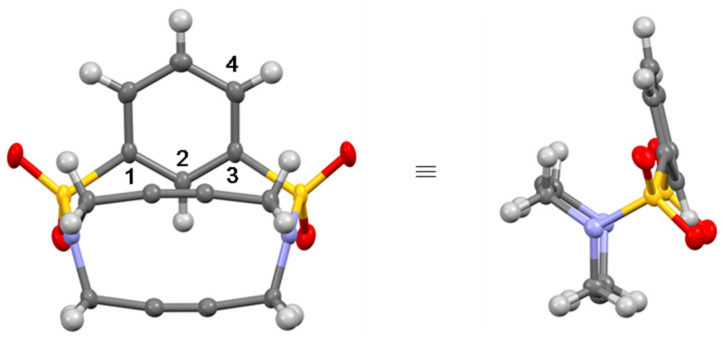
Crystal graphic of compound **8** (CCDC No. 2417595).

Both compounds **7** and **8** were successfully recrystallized using organic solvents, and their structures were elucidated via X-ray analysis, as presented in Figure 4 and Figure 5.

Table 1 summarizes the structural parameters obtained from crystallographic analysis of compounds **12**, **7** and **8**. A key conformational difference lies in the distance between the nitrogen atoms of the two sulfonamide bonds. In compound **12**, this distance is 6.96 Å, while in compound **7**, it decreases to 5.33 Å. Upon formation of the second butyne tether in compound **8**, the nitrogen–nitrogen distance further decreases to 4.88 Å. In contrast, the distance between sulfur atoms exhibits minimal change. In compound **12**, which lacks a ring, and compound **7**, which contains a single butyne ring, the sulfur–sulfur distance remains at 5.41 Å. However, in compound **8**, which features a dibutyne ring, the sulfur–sulfur distance slightly decreases to 5.30 Å. The angle formed by the benzene ring and the sulfur–nitrogen bond is also noteworthy, bending from 108° to 105°. The angle of the alkyne moieties in compounds **7** and **8** is bent by approximately 5–10°, and a distorted angle originating from the middle ring alkyne is observed. The bond lengths of the alkynes remain consistent. Comparing the distortion of the benzene moiety in compounds **7** and **8**, there is negligible distortion in the dihedral angle formed by C1 to C4. However, the dihedral angle formed by C1 to C4 is 4.56° for alkyne **7** and 5.27° for dialkyl **8**, indicating that molecular distortion impacts the benzene moiety. Furthermore, the sulfur atoms gradually shift out of the plane formed by the benzene ring, as evidenced by the torsional angle C1–C2–C3–S changing from 177° to 170° to 162°. This deviation becomes more pronounced with the addition of a butyne ring. Although a significant difference is observed in the proton NMR spectra, the distance between the 2nd position of hydrogen of the benzene ring and the alkyne carbon atom remains unchanged between compounds **7** and **8** in the crystalline state.

### 2.4. Proton NMR Data Comparison

**Figure 6:**
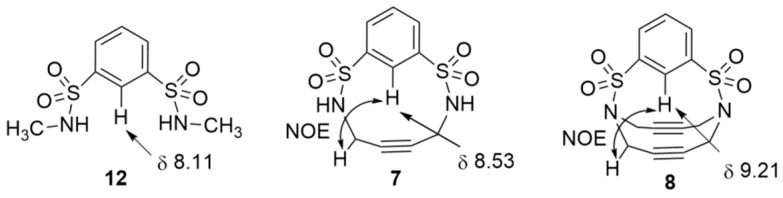
Structure and ^1^H-NMR chemical shift relation.

**Figure 7:**
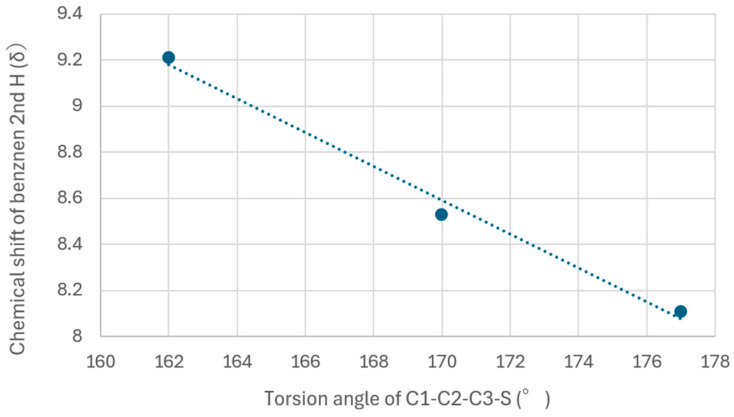
Correlation between chemical shift and torsion angle.

The ^1^H NMR chemical shift of the hydrogen atom at the second position of the benzene ring in DMSO-*d*_6_ and the corresponding NOESY spectrum indicates a Csp^2^-H to alkyne π close interaction (Figure 6). The spectra of NMR chart see the Supplementary Materials. However, there is no difference in the distance between the hydrogen atom and the alkyne group in the crystals of Compound **7** (2.82 Å) and Compound **8** (2.83 Å). The order of these chemical shift values, δ8.11 (**12**) < 8.53 (**7**) < 9.21 (**8**), is inversely proportional to their torsion angle (°): C1-C2-C3-S 177 > 170 > 162 (Figure 7).

It is inferred that the displacement of the sulfur atom from the benzene ring, caused by the strain of the 11-membered ring, influences the proton shift.

### 2.5. Carbon NMR Data Comparison

**Figure 8:**
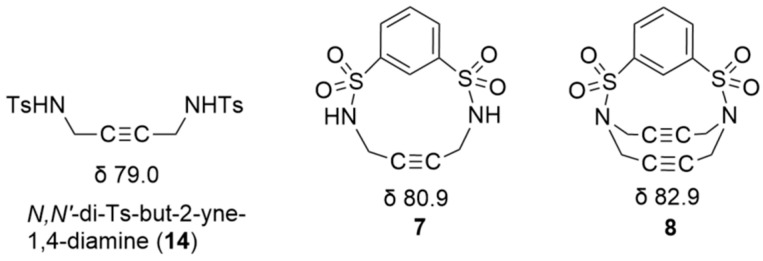
Alkyne and ^13^C-NMR chemical shift relation.

**Figure 9:**
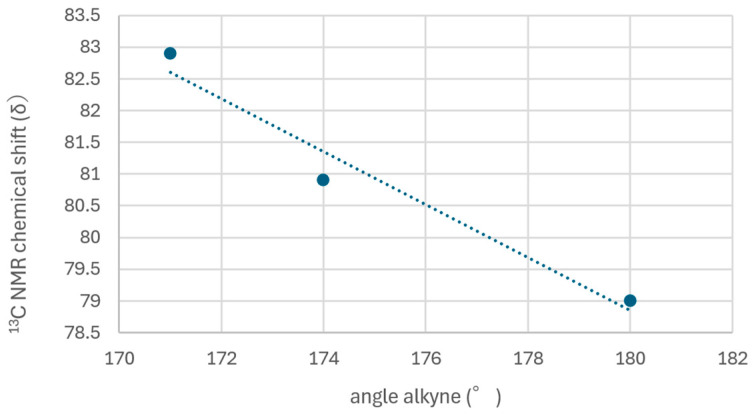
Correlation between alkyne angle and chemical shift.

The ^13^C NMR chemical shifts of the alkyne carbons are shown in Figure 8. It is known that bond angle strain in alkyne carbons affects their ^13^C NMR chemical shifts [16]. Therefore, we compared the -C-C≡C- angle and ^13^C NMR chemical shift value in *d*_6_-DMSO solution using the standard compound *N*,*N*’-bis-*p*-toluenesulfonyl-but-2-yne-1,4-diamine (**14**) [22]. Compared with the normal alkyne angle at δ79.0 in ^13^C NMR chemical shift value in DMSO-*d*_6_ solution, **7** showed a 173° angle at δ80.9 and **8** showed a 171° angle at δ82.9, respectively (Figure 9). The distortion order observed in the crystal structure, **8** > **7** > **14**, may be detectable in organic solvent solutions, and the ^13^C NMR chemical shifts provide a useful method to estimate alkyne bond angle distortions.

### 2.6. Comparison of DFT Calculations with Experimental Data

Based on structures obtained via X-ray crystallography, theoretical NMR values in solution were calculated using density functional theory (DFT) [23,24,25] (Table 2). The detailed DFT calculation methods see the Supplementary Materials. Although systematic errors were observed between the theoretical and experimental values (approximately +0.5–0.6 ppm for ^1^H and +7 ppm for ^13^C), the consistency of this offset across the three compounds indicates good relative reproducibility between the calculated and measured values. It can be concluded that the number of alkyne linkages introduced restricts the conformational freedom of the ring, and the resulting changes in structural and electronic strain—accompanying the progressive rigidification of the ring—manifest as a quantitative downfield shift in the NMR signals observed in solution.

## 3. Materials and Methods

### 3.1. General Remarks

All organic reactions were typically conducted in general glassware under an appropriate atmosphere. All reagents were used as purchased from commercial sources without further purification. All solvents were used as purchased from commercial sources without additional distillation. Flash chromatography was performed on Kanto silica gel 60N (spherical, neutral, 40−50 μm). Melting points were measured using a Yanaco micro-melting point apparatus. ^1^H and ^13^C NMR spectra were recorded using JEOL ECA 600 or JEOL ECZ 400 spectrometers (Tokyo, Japan) in deuterated solvent. The chemical shifts were reported in ppm (δ) using tetramethylsilane (TMS) or deuterated solvent as an internal control. Infrared spectra were obtained with Shimazu IR-Affinity 1S spectrometers (Kyoto, Japan). High-resolution mass spectrometry (HRMS) analyses were performed using a JEOL JMS-600, JEOL JMS-T100GCv, or Thermo Scientific Exactive mass spectrometer (Waltham, MA, USA). X-ray crystallography structural analyses were performed using a Bruker SMART APEX II ULTRA diffractometer (Billerica, MA, USA).

### 3.2. Experimental Procedures and Characterization of New Compounds

#### 3.2.1. Di-tert-butyl (1,3-Phenylenedisulphonyl) Dicarbamate (**10**)

A total of 2.36 g of **9** (10 mmol) was dissolved in 40 mL of dried CH_2_Cl_2_ under an argon atmosphere, followed by the addition of 4.82 mL of di-*tert*-butyl dicarbonate (Boc_2_O, 21 mmol) and 3.46 mL of triethylamine (Et_3_N, 25 mmol) at room temperature. The mixture was stirred vigorously. Then, 122 mg of 4-dimethylaminopyridine (DMAP, 1.0 mmol) was added to the reaction mixture, which was stirred for 24 h at room temperature. After 24 h, 40 mL of 1 mol/L aqueous HCl solution was added to the mixture, followed by stirring for 10 min. Two layers formed and were separated. The aqueous layer was extracted with 40 mL of CH_2_Cl_2_ two times. The combined CH_2_Cl_2_ layers were washed with brine and dried over MgSO_4_. The mixture was filtered to remove the MgSO_4_ and the filtrate evaporated to remove the CH_2_Cl_2_. The sticky residue was purified by silica gel column chromatography by eluting with n-hexane:AcOEt (1:1) to give 4.15 g (95% yield) of **10** as a white solid.

Mp 139–140 °C; ^1^H NMR (600 MHz, CDCl_3_) δ 8.58 (s, 1H), 8.32 (d, *J* = 8.3 Hz, 2H), 7.76 (t, *J* = 8.3 Hz, 1H), 7.60–7.45 (br s, 2H), 1.39 (s, 18H); ^13^C NMR (151 MHz, CDCl_3_) δ 148.7, 140.3, 133.5, 129.8, 127.7, 85.1, 27.9; IR (neat) 3176, 3103, 2971, 1698, 1363, 1148 cm^−1^; HRMS (ESI^+^) *m*/*z* [M + Na]^+^ calcd for C_16_H_24_N_2_O_8_S_2_Na: 459.0866; found 459.0865.

#### 3.2.2. Di-tert-butyl (1,3-Phenylenedisulfonyl)bis(methylcarbamate) (**11**)

Dimethoxyethyl azodicarboxylate (DMEAD, 491 mg, 2.1 mmol) and dehydrated methanol (85 μL, 2.1 mmol) were added to a solution containing compound **10** (436 mg, 1.0 mmol) and triphenyl phosphine (TPP, 550 mg, 2.1 mmol) in 20 mL of anhydrous THF under an argon atmosphere at room temperature. The mixture was stirred for 24 h, and then the reaction mixture was concentrated under vacuum. The residue was dissolved in 20 mL of AcOEt and water (1:1) and subjected to two extractions. The organic layer obtained was washed with brine and dried over MgSO_4_. The dried solution was filtered to remove MgSO_4_, and the filtrate was evaporated under vacuum. The resulting residue was purified using silica gel column chromatography by eluting with n-hexane:AcOEt (5:1 to 3:1) to give 396 mg (85% yield) of **11** as a white solid.

Mp 131–132 °C; ^1^H NMR (400 MHz, CDCl_3_) δ 1.38 (s, 18H), 3.37 (s, 6H), 7.69 (t, *J* = 8.4 Hz, 1H), 8.14 (dd, *J* = 1.6, 8.4 Hz, 2H), 8.40 (t, *J* = 1.6 Hz, 1H); ^13^C NMR (100 MHz, CDCl_3_) δ 27.9, 33.6, 85.3, 127.2, 129.5, 132.3, 141.2, 150.8; IR (neat) 1742, 1359, 1290, 1152 cm^−1^; HRMS (ESI^+^) *m*/*z* [M + Na]^+^ calcd for C_18_H_28_N_2_O_8_S_2_Na: 487.1179; found 487.1167.

#### 3.2.3. N,N′-Dimethylbenzene-1,3-disulfonamide (**12**)

Trifluoroacetic acid (TFA, 0.39 mL, 5.0 mmol) was added to the solution of **11** (234 mg, 0.504 mmol) in 5.0 mL of tetrahydrofuran (THF) at room temperature. The mixture was stirred for 24 h, and then the reaction mixture was concentrated under reduced pressure. The residue was filtered using diethyl ether, and the obtained solid was dried under vacuum. In total, 83.3 mg of the desired product **12** (63% yield) was obtained as a white solid. This was dissolved in AcOEt, and the solution was treated with a few drops of *n*-hexane and left at room temperature. The desired colorless single crystal was obtained after several days.

Mp 125 °C, ^1^H NMR (400 MHz, DMSO-*d*_6_) δ8.10 (s, 1H, benzene 2nd H), 8.00 (dd, *J* = 1.6, 7.6 Hz, 2H, benzene 4,6th H), 7.83 (t, *J* = 7.6 Hz, 1H, benzene 5th H), 7.71 (q, *J* = 5.2 Hz, 2H, N-H), 2.41 (d, *J* = 5.2 Hz, 6H, -CH_3_), ^13^C NMR (100 MHz, DMSO-*d*_6_) δ 140.9, 131.3, 130.9, 125.2, 29.2, IR (neat) 3270, 1414, 1321, 1130, 1063 cm^−1^, HRMS (ESI^+^): *m*/*z* [M + Na]^+^ calcd for C_8_H_12_O_4_N_2_NaS_2_: 287.0131; found 287.0129.

#### 3.2.4. N,N’-Di-tert-butoxycarbonyl-1,3-benzenedisulfonamide-containing Cycloundecyne (**13**)

DMEAD (66.5 mg, 0.289 mmol) was added to a solution of **10** (50 mg, 0.115 mmol), but-2-yne-1,4-diol (9.89 mg, 0.115 mmol), and DMAP-DPP (92.3 mg, 0.289 mmol) in 11.5 mL of anhydrous THF under an argon atmosphere at room temperature. After stirring for 24 h, the reaction mixture was concentrated *in vacuo*. The residue was dissolved in 12 mL of AcOEt and extracted with 12 mL of 1 mol/L aqueous HCl solution. The organic layer obtained was washed with H_2_O and brine, and dried over MgSO_4_. The solution was filtered to remove MgSO_4_, and the filtrate evaporated under vacuum. The residue was purified by silica gel column chromatography by eluting with *n*-hexane:AcOEt (5:1) to give 47.2 mg (84% yield) of white solid **13**.

Mp 179–181 °C; ^1^H NMR (400 MHz, CDCl_3_) δ 9.21 (s, 1H), 8.24 (dd, *J* = 7.9, 1.7 Hz, 2H), 7.66 (t, *J* = 7.9 Hz, 1H), 4.65 (s, 4H), 1.36 (s, 18H); ^13^C NMR (100 MHz, CDCl_3_) δ 149.8, 139.4, 133.7, 129.3, 127.5, 85.6, 80.0, 37.5, 27.9; IR (neat) 1724, 1382, 1148 cm^−1^; HRMS (ESI^+^) *m*/*z* [M + Na]^+^ calcd for C_20_H_26_N_2_O_8_S_2_Na: 509.1023; found 509.1024.

#### 3.2.5. 1,3-Benzene-di-sulfonamide-containing Cycloundecyne (DBSACU, **7**)

Trifluoroacetic acid (TFA, 18 mL, 237 mmol) was added to the solution of **13** (2.30 g, 4.73 mmol) in 22 mL of CH_2_Cl_2_ at room temperature. The mixture gradually became a cloudy suspension while being stirred. After 5 h, the suspension was concentrated under reduced pressure to remove CH_2_Cl_2_ and the remaining TFA. The residue was filtered using diethyl ether and water to obtain a white solid, which was dried under vacuum. In total, 1.19 g of **7** (88% yield) was obtained as a pure white powder, which was dissolved in hot acetone and left at room temperature for 10 days. The desired colorless single crystal was obtained.

MP decomp over 285 °C, ^1^H-NMR (600 MHz, DMSO-*d*_6_) δ 8.53 (s, 1H, benzene 2nd H), 8.11 (t, *J* = 6.0 Hz, 2H, benzene 4,6th H), 8.06–7.97 (m, 2H, N-H), 7.77 (t, *J* = 7.9 Hz, 1H, benzene 5th H), 3.80 (d, *J* = 6.0 Hz, 4H, -CH_2_-), ^13^C NMR (150 MHz, DMSO-*d*_6_) δ 141.0, 130.99, 130.91, 128.4, 80.9, 33.0, IR (neat) 3269, 1434, 1326, 1150 cm^−1^, HRMS (ESI^−^): *m*/*z* [M − H]^−^ calcd for C_10_H_9_O_4_N_2_S_2_: 285.0009; found 285.0014.

#### 3.2.6. Benzene-1,3-disulfonamide Containing Dicycloundecyne (BDSADCU, **8**)

Dimethoxyethyl azodicarboxylate (DMEAD, 491 mg, 2.1 mmol) and but-2-yn-1,4-diole (86, 1.0 mmol) were added to a solution of **7** (286 mg, 1.0 mmol) and 4-dimethylaminophenyl diphenylphosphine (DMAP-DPP, 640 mg, 2.1 mmol) in 10 mL of anhydrous THF under an argon atmosphere at room temperature. The mixture was stirred for 17 h, and then the reaction mixture was concentrated under vacuum. The residue was rinsed with 1 mol/L hydrochloric acid. The resulting residue was purified using silica gel column chromatography with chloroform to give 62.8 mg (18.6% yield) of **8** as a white solid. Then, **8** was recrystallized from hot 1,4-dioxane.

MP decomp over 230 °C, ^1^H-NMR (400 MHz, DMSO-*d*_6_) δ 9.21 (s, 1H, benzene 2nd H), 8.13 (d, *J* = 8.0 Hz, 2H, benzene 4,6th H), 7.86 (t, *J* = 8.0 Hz, 1H, benzene 5th H), 4.21–4.08 (m, 8H, -CH_2_-), ^13^C-NMR (100 MHz, DMSO-d6) δ139.8, 134.1, 131.5, 130.3, 82.9, 40.0, IR (neat) 1444, 1332, 1152 cm^−1^, HRMS (ESI^+^): *m*/*z* [M + Na] ^+^calcd for C_14_H_12_N_2_O_4_S_2_Na: 359.0131; found 359.0133.

## 4. Conclusions

In summary, we synthesized BDSACU **7** and BDSADCU **8**, novel heterocyclic molecules containing benzene, two sulfonamides and alkynes. X-ray crystal structures showed flexibility in the sulfur–nitrogen bond length and of the carbon–sulfur–nitrogen angle in the sulfonamide state, deviation from the planar structure of the benzene moiety, and angle distortion from the straight-line structure of the alkyne moiety. In the ^1^H NMR measurements in DMSO solution, characteristic chemical shifts of hydrogen atoms were observed, which were correlated to the dihedral angle involving the benzene ring and sulfonamide sulfur in the crystal state. Furthermore, in the ^13^C NMR spectra, a correlation between the alkyne bond angle and the chemical shifts was confirmed. The observed relative chemical shift changes among these compounds are in quantitative agreement with those calculated via DFT based on the crystal structures, supporting the correlation between the crystal structures and the NMR values. In conclusion, analyses using crystal parameters, DFT, and NMR in DMSO solution revealed that these newly designed molecules exhibit conformational distortions arising from the strain imposed by the 11-membered ring. This research lays the groundwork for the design of sulfonamide-containing medium-sized ring molecules and the development of sulfonamide drugs.

## Figures and Tables

**Table 1:** Comparison of structural data from crystallographic analysis.

	12	7	8
N-N distance (Å)	6.96	5.33	4.88
S-S distance (Å)	5.41	5.41	5.31
Angle Csp^2^-S-N (°)	108	108	105
Alkyne angle (°)	-	173–175	171
Length S-N (Å)	1.61	1.61	1.64
Length Csp≡Csp (Å)	-	1.19	1.20
Length C-S (Å)	1.77	1.77	1.77
Angle O-S-O (°)	120	119–120	120
Torsion angle Benzene C1-C2-C3-C4 (°)	0.36	4.54	5.27
Torsion angle C1-C2-C3-S (°)	177	170	162
Benzene 2nd H-Alkyne Csp distance (Å)	-	2.82	2.83

**Table 2:** Comparison of calculated and measured values.

	Calculated Value δ	Measured Value δ	Δδ (Calculated–Measured)
Compound **12** (^1^H)	8.713	8.11	+0.603
Compound **7** (^1^H)	9.062	8.53	+0.532
Compound **8** (^1^H)	9.832	9.21	+0.622
Compound **7** (^13^C)	87.948	80.9	+7.048
Compound **8** (^13^C)	90.205	82.9	+7.305

## Data Availability

Crystallographic data for compounds **7**, **8**, and **12** has been deposited at the CCDC under Nos. 2402073, 2417595, and 2402057 and can be obtained from https://www.ccdc.cam.ac.uk, accessed on 11 July 2026.

## Supplementary Materials

The following supporting information can be downloaded at https://www.mdpi.com/article/10.3390/molecules31142462/s1. Refs. [25,26,27,28,29,30] are cited in Supplementary Materials.
