# Supplementary material for: Conformational Analysis of Novel Benzene-1,3-Disulfonamide-Based Cycloalkynes Through X-Ray Crystallography, DFT Calculations, and NMR Spectroscopy
Source: Molecules. 2026 Jul 14;31(14):2462. doi: 10.3390/molecules31142462 (PMC13414055; doi:10.3390/molecules31142462)
Supplement: Supplementary file 1 [file molecules-31-02462-s001.zip › Supporting Information (Computational).pdf]

Computational Details

Conformational searches for compounds **7**, **8**, and **12** were conducted using the CREST[1)] program based on the semiempirical GFN2-xTB[2)] method, employing the ALPB implicit solvation model for dimethyl sulfoxide (DMSO). Conformers within an energy window of 5.0 kcal/mol were extracted and subjected to geometry optimization using the ORCA[3)] 6.1.0 program package. Initial pre-optimizations were performed at the B3LYP/def2-SVP level. Subsequently, the selected low-energy conformers were further optimized at the ωB97X-D3/def2-TZVP level. Harmonic vibrational frequency calculations were performed at the identical level of theory to confirm that all optimized structures are true energy minima (absence of imaginary frequencies) and to obtain the thermal corrections to Gibbs free energies at 298.15 K. The SMD implicit solvation model (DMSO) was applied in all DFT calculations. The relative populations of the conformers in solution were determined based on their relative Gibbs free energies using the Boltzmann distribution.

The isotropic nuclear magnetic shielding constants (σ) of the conformers were calculated using the Gauge-Independent Atomic Orbital (GIAO) method at the ωB97X-D3/def2-TZVP level with the SMD(DMSO) model. The theoretical chemical shifts (δ_{calc}) were obtained by subtracting the population-weighted average of the shielding constants (δ_{sample}) from the corresponding shielding constants of tetramethylsilane (δ_{TMS}), which were calculated at the identical level of theory (δ_{calc} = δ_{TMS} - δ_{sample}). The calculated reference values for δ_{TMS} were 31.834 ppm for ^{1}H and 191.592 ppm for ^{13}C. Molecular structures and theoretical parameters were analyzed and visualized using the Avogadro[4)] software.

References

1) Pracht, P.; Bohle, F.; Grimme, S. Automated exploration of the low-energy chemical space with fast quantum chemical methods. *Phys. Chem. Chem. Phys.* **2020**, *22*, 7169–7192.

2) Bannwarth, C.; Ehlert, S.; Grimme, S. GFN2-xTB—An Accurate and Broadly Parametrized Self-Consistent Tight-Binding Quantum Chemical Method with Multipole Polarization and Density Dependent Dispersion Contributions. *J. Chem. Theory Comput.* **2019**, *15*, 1652–1671.

3) Neese, F. Software update: The ORCA program system—Version 5.0. *WIREs Comput. Mol. Sci.* **2022**, *12*, e1606.

4) Hanwell, M. D.; Curtis, D. E.; Lonie, D. C.; Vandermeersch, T.; Zurek, E.; Hutchison, G. R. Avogadro: an advanced semantic chemical editor, visualization, and analysis platform. *J. Cheminform.* **2012**, *4*, 17.
