# Supplementary material for: Conformational Analysis of Novel Benzene-1,3-Disulfonamide-Based Cycloalkynes Through X-Ray Crystallography, DFT Calculations, and NMR Spectroscopy
Source: Molecules. 2026 Jul 14;31(14):2462. doi: 10.3390/molecules31142462 (PMC13414055; doi:10.3390/molecules31142462)

Supporting Information for

Conformational Analysis of Novel Benzene-1,3-disulfonamide-Based Cycloalkynes through X-ray Crystallography, DFT Calculations, and NMR Spectroscopy

Kyosuke Kaneda*, Takato Koideya , Hitomi Tsuda, Haruto Katakura, Haruhiko Fukaya and Takehiro Yamagishi

^{1}H-NMR (400 MHz, DMSO-d6) spectrum of **12**

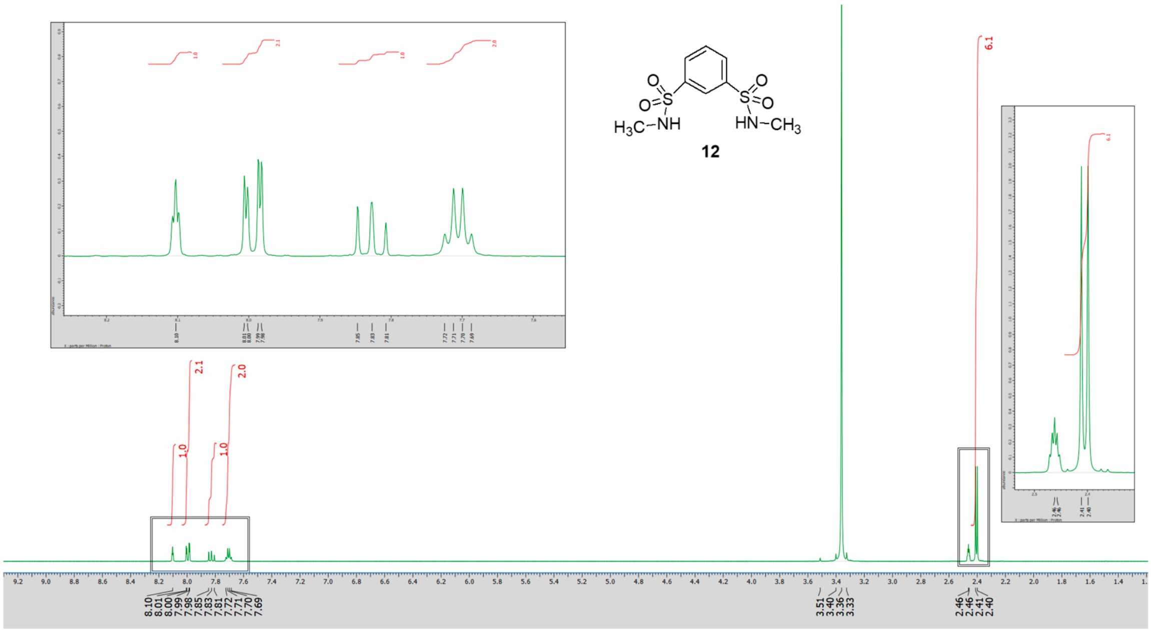

^{13}C-NMR (100 MHz, DMSO-d6) spectrum of **12**

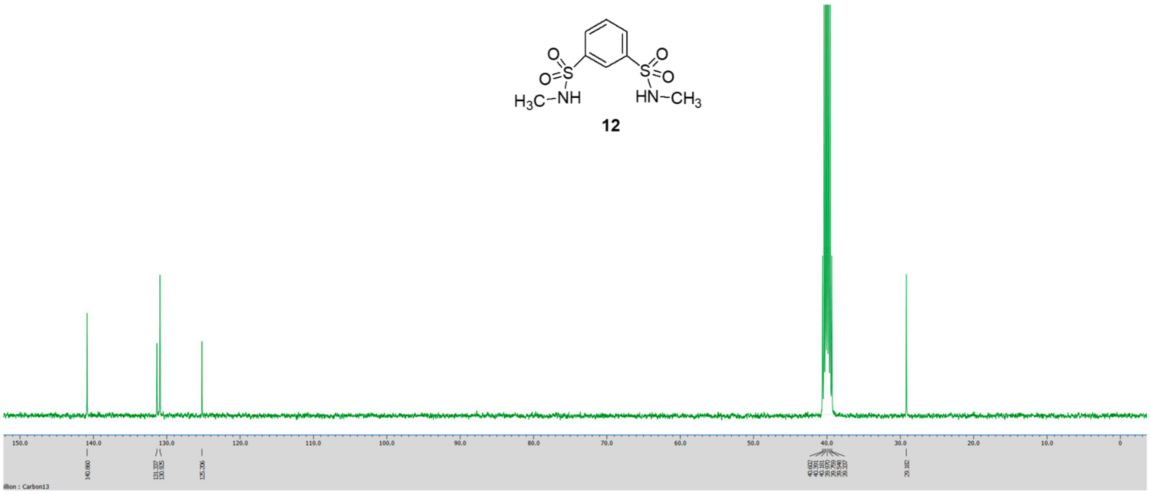

^{1}H-NMR (600 MHz, DMSO-*d6*) spectrum of **7**

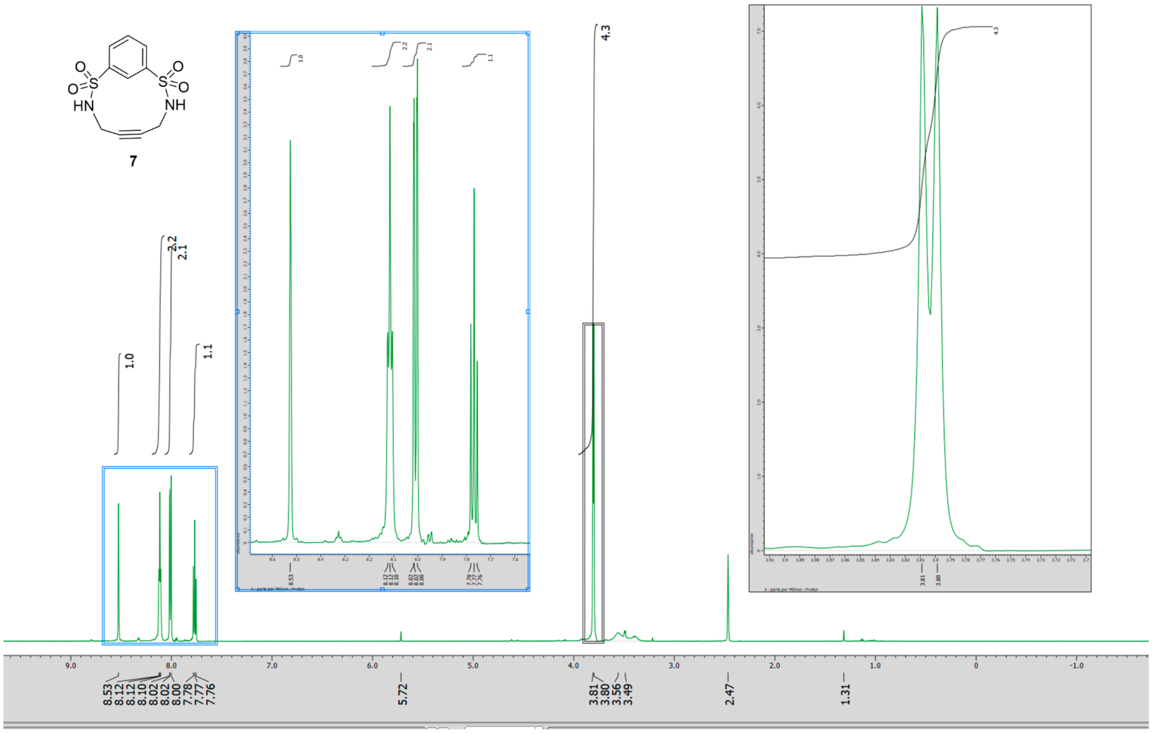

^{13}C-NMR (150 MHz, DMSO-*d6*) spectrum of **7**

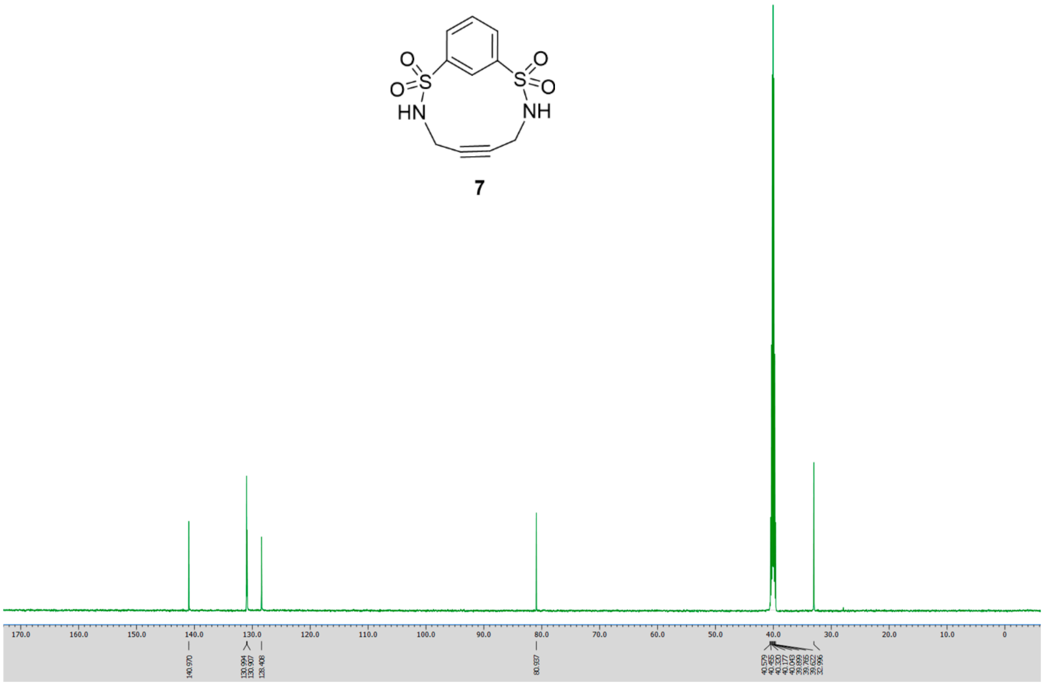

NOESY spectrum of compound **7** (DMSO-*d6*)

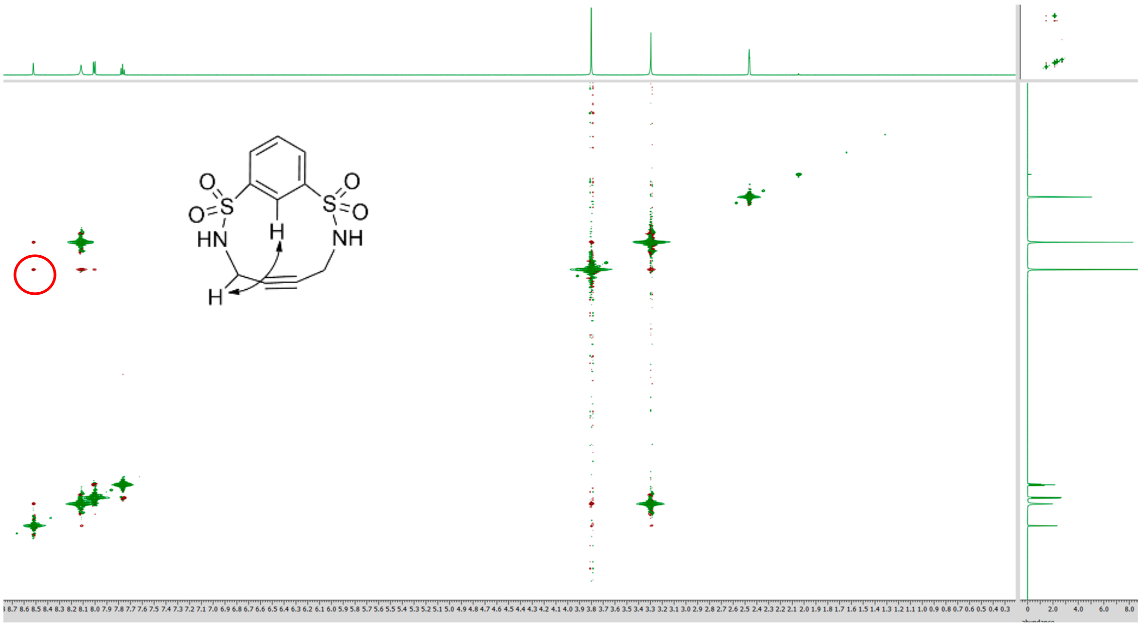

^{1}H-NMR (400 MHz, DMSO-*d6*) spectrum of **8**

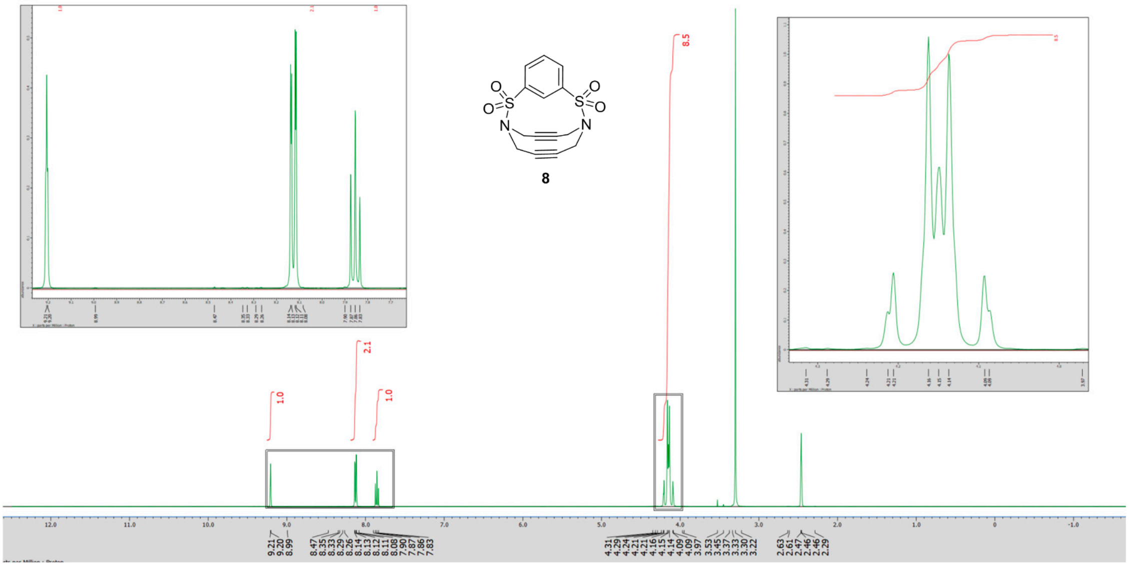

^{13}C-NMR (100 MHz, DMSO-*d6*) spectrum of **8**

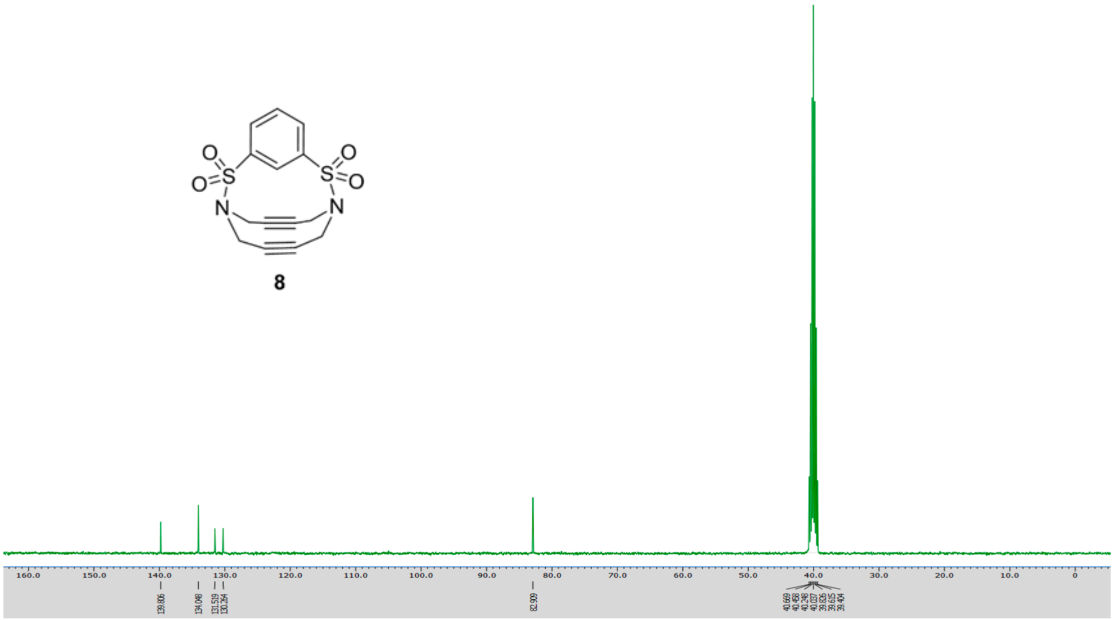

NOESY spectrum of compound **8** (DMSO-*d6*)

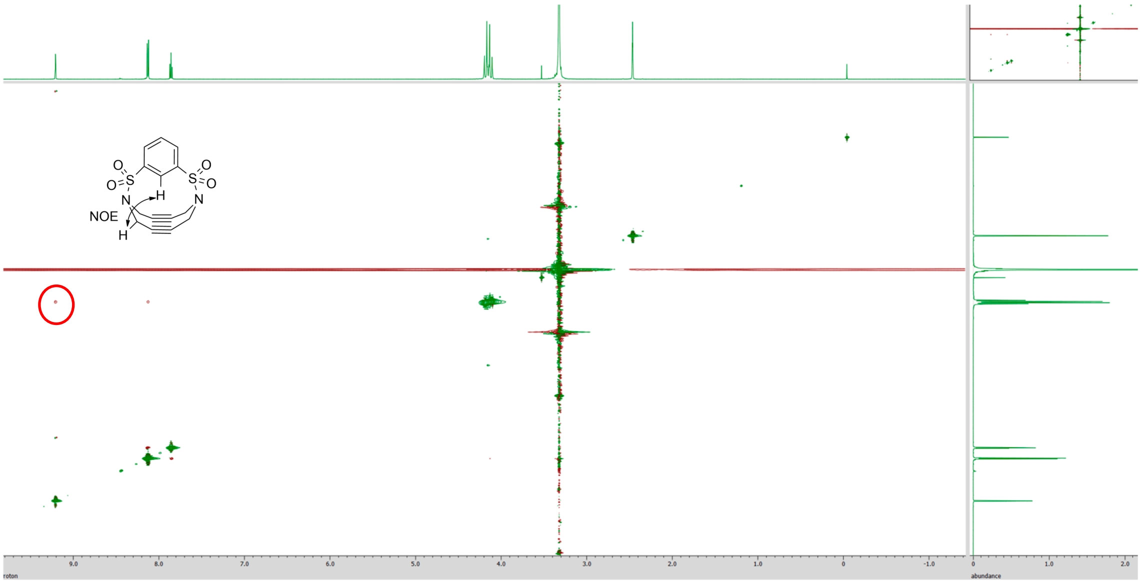

Supplement: Supplementary file 1 [file molecules-31-02462-s001.zip › Supporting Information [NMR].pdf]
