# Supplementary material for: Conformational Analysis of Novel Benzene-1,3-Disulfonamide-Based Cycloalkynes Through X-Ray Crystallography, DFT Calculations, and NMR Spectroscopy
Source: Molecules. 2026 Jul 14;31(14):2462. doi: 10.3390/molecules31142462 (PMC13414055; doi:10.3390/molecules31142462)
Supplement: Supplementary file 1 [file molecules-31-02462-s001.zip › X-ray crystallography data of compound 12.pdf]

X-ray crystallography data of compound 12

R_1=4.60%

Crystal Data and Experimental

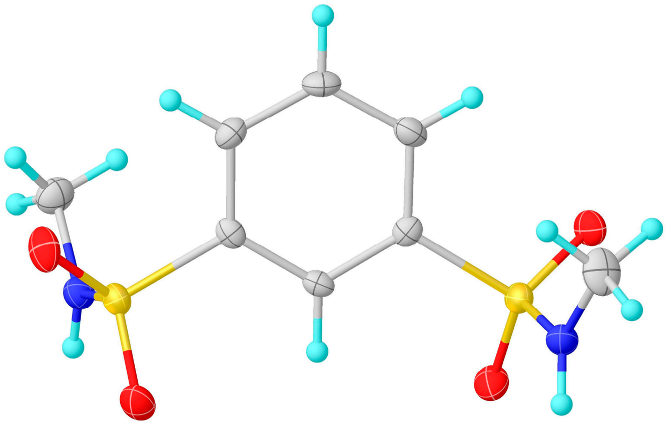

Experimental. Single colourless block-shaped crystals of **12** were obtained from slow evaporation of ethyl acetate at room temperature. A suitable crystal 0.17×0.14×0.04 mm³ was selected and mounted on a MiTeGEN Dual Thickness MicroLoops in perfluoropolyether oil on an XtaLAB Synergy R, HyPix diffractometer. The crystal was kept at a steady *T* = 99.9(3) K during data collection. The structure was solved with the ShelXT 2018/2 (Sheldrick, 2018) structure solution program using the Intrinsic Phasing solution method and by using **Olex2** (Dolomanov et al., 2009) as the graphical interface. The model was refined with version 2018/3 of ShelXL 2018/3 (Sheldrick, 2015) using Least Squares minimisation.

Crystal Data. $C_8H_{12}N_2O_4S_2$, M_r = 264.32, orthorhombic, $P2_12_12_1$ (No. 19), a = 8.15450(10) Å, b = 20.6728(2) Å, c = 13.78410(10) Å, $\alpha = \beta = \gamma$ = 90°, V = 2323.67(4) Å³, T = 99.9(3) K, Z = 8, Z' = 2, μ(Cu K$_\alpha$) = 4.210, 21476 reflections measured, 4781 unique (R_{int} = 0.0371) which were used in all calculations. The final wR_2 was 0.1312 (all data) and R_1 was 0.0460 (I > 2(I)).

ORTEP drawing of **12** showing thermal ellipsoids at the 50% probability level.

**Compound**	**12**
Formula	$C_8H_{12}N_2O_4S_2$
$D_{calc.}$/ g cm^{-3}	1.511
μ/mm^{-1}	4.210
Formula Weight	264.32
Colour	colourless
Shape	block
Size/mm³	0.17×0.14×0.04
T/K	99.9(3)
Crystal System	orthorhombic
Flack Parameter	0.078(10)
Hooft Parameter	0.077(7)
Space Group	$P2_12_12_1$
a/Å	8.15450(10)
b/Å	20.6728(2)
c/Å	13.78410(10)
α/°	90
β/°	90
γ/°	90
V/Å³	2323.67(4)
Z	8
Z'	2
Wavelength/Å	1.54184
Radiation type	Cu K$_\alpha$
Θ_{min}/°	3.854
Θ_{max}/°	77.655
Measured Refl.	21476
Independent Refl.	4781
Reflections with I > 2(I)	4533
R_{int}	0.0371
Parameters	293
Restraints	0
Largest Peak	0.733
Deepest Hole	-0.579
GooF	1.070
wR_2 (all data)	0.1312
wR_2	0.1298
R_1 (all data)	0.0480
R_1	0.0460

Structure Quality Indicators

**Reflections:**	d min (Cu\a) 2Θ=155.3° 0.79	I/σ(I) 41.2	Rint 3.71%	Full 135.4° 98% to 155.3° 99.8	
**Refinement:**	Shift 0.000	Max Peak 0.7	Min Peak -0.6	GooF 1.070	Hooft .077(7)

Experimental Extended. A colourless block-shaped crystal with dimensions 0.17×0.14×0.04 mm^3 was mounted on a MiTeGEN Dual Thickness MicroLoops in perfluoropolyether oil. Data were collected using an XtaLAB Synergy R, HyPix diffractometer operating at T = 99.9(3) K.

Data were measured using ω scans of 0.5° per frame for 0.1/0.3 s using Cu K_α radiation. The diffraction pattern was indexed and the total number of runs and images was based on the strategy calculation from the program CrysAlisPro (Rigaku, V1.171.42.84a, 2023) The maximum resolution that was achieved was Θ = 77.655° (0.79 Å).

The diffraction pattern was indexed The diffraction pattern was indexed and the total number of runs and images was based on the strategy calculation from the program CrysAlisPro (Rigaku, V1.171.42.84a, 2023) and the unit cell was refined using CrysAlisPro (Rigaku, V1.171.42.84a, 2023) on 13988 reflections, 65% of the observed reflections.

Data reduction, scaling and absorption corrections were performed using CrysAlisPro (Rigaku, V1.171.42.84a, 2023). The final completeness is 99.60 % out to 77.655° in Θ. A multi-scan absorption correction was performed using CrysAlisPro 1.171.42.84a (Rigaku Oxford Diffraction, 2023) using spherical harmonics,implemented in SCALE3 ABSPACK scaling algorithm. The absorption coefficient μ of this material is 4.210 mm^{-1} at this wavelength (λ = 1.542Å) and the minimum and maximum transmissions are 0.701 and 1.000.

The structure was solved and the space group $P2_12_12_1$ (# 19) determined by the ShelXT 2018/2 (Sheldrick, 2018) structure solution program using Intrinsic Phasing and refined by Least Squares using version 2018/3 of ShelXL 2018/3 (Sheldrick, 2015). All non-hydrogen atoms were refined anisotropically. Hydrogen atom positions were calculated geometrically and refined using the riding model. Hydrogen atom positions were calculated geometrically and refined using the riding model.

_exptl_absorpt_process_details: CrysAlisPro 1.171.42.84a (Rigaku Oxford Diffraction, 2023) using spherical harmonics,implemented in SCALE3 ABSPACK scaling algorithm.

The Flack parameter was refined to 0.078(10). Determination of absolute structure using Bayesian statistics on Bijvoet differences using the Olex2 results in 0.077(7). Note: The Flack parameter is used to determine chirality of the crystal studied, the value should be near 0, a value of 1 means that the stereochemistry is wrong and the model should be inverted. A value of 0.5 means that the crystal consists of a racemic mixture of the two enantiomers.

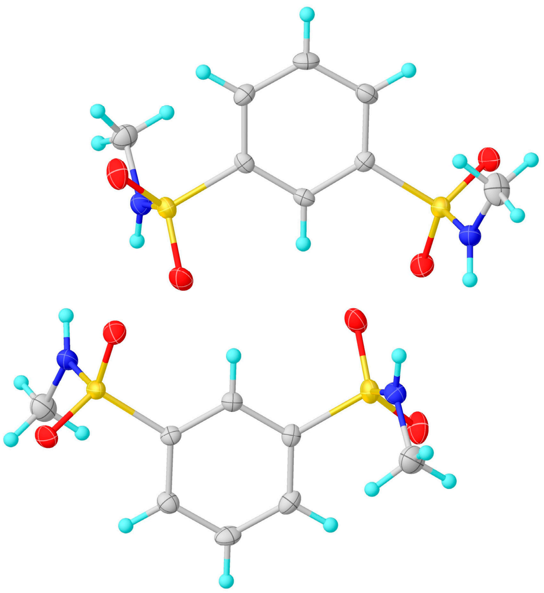

ORTEP diagram of the asymmetric unit showing two molecules of **12** with 50% probability ellipsoids **.**

Table S13: Fractional Atomic Coordinates (×10⁴) and Equivalent Isotropic Displacement Parameters (Å²×10³) for **12**. U_{eq} is defined as 1/3 of the trace of the orthogonalised U_{ij}.

Atom	x	y	z	U_{eq}
C1	8286(5)	5446(2)	2290(4)	16.7(9)
C2	7486(5)	4945(2)	2767(4)	16.3(10)
C3	6885(5)	4436(2)	2208(4)	16.2(9)
C4	7086(6)	4419(2)	1204(4)	20.5(10)
C5	7909(7)	4926(2)	758(4)	23.2(10)
C6	8515(6)	5443(3)	1296(4)	20.7(10)
C7	7197(7)	7004(3)	2134(4)	30.7(12)
C8	8280(7)	2954(3)	2450(4)	27.3(11)
N1	7583(5)	6647(2)	3017(3)	21.1(9)
N2	7278(5)	3283.1(19)	3178(3)	20.4(9)
O1	10409(4)	6371.3(17)	2544(3)	23.7(8)
O2	9139(5)	5870.9(17)	3995(3)	25.5(8)
O3	4879(4)	3462.4(18)	2130(3)	28.2(8)
O4	5162(4)	4050.1(18)	3686(3)	26.8(8)
S1	8994.9(14)	6101.9(5)	3015.7(8)	18.1(3)
S2	5898.4(14)	3788.9(5)	2819.1(9)	18.6(3)
C9	2970(6)	5543(2)	5287(4)	17.2(9)
C10	2828(6)	5534(2)	6291(4)	21.5(10)
C11	2036(7)	5021(2)	6741(4)	23.3(10)
C12	1391(6)	4519(3)	6185(4)	20.2(10)
C13	1559(5)	4533(2)	5183(4)	16.0(9)
C14	2343(6)	5044(2)	4732(4)	17.7(10)
C15	1579(7)	7035(3)	5021(5)	31.8(13)
C16	2715(7)	3013(3)	5346(4)	30.0(12)
N3	2614(5)	6696(2)	4311(3)	19.2(9)
N4	2291(5)	3347(2)	4436(3)	22.0(9)
O5	4743(4)	5933.6(18)	3839(3)	28.0(8)
O6	4947(5)	6516.5(17)	5414(3)	30.0(9)
O7	699(5)	4122.6(17)	3485(3)	24.8(8)
O8	-541(4)	3604.5(17)	4921(3)	24.6(8)
S3	3973.9(14)	6193.9(5)	4695.7(9)	19.4(3)
S4	861.1(14)	3887.4(5)	4456.3(8)	17.4(3)

Table S14: Anisotropic Displacement Parameters (×10[4]) **12.** The anisotropic displacement factor exponent takes the form: $-2\pi^2[h^2a^{*2} \times U_{11} + ... + 2hka^* \times b^* \times U_{12}]$

Atom	U_{11}	U_{22}	U_{33}	U_{23}	U_{13}	U_{12}
C1	14(2)	17(2)	19(2)	-2.2(18)	-1.3(18)	1.6(17)
C2	15(2)	20(2)	14(2)	2.3(17)	1.4(16)	0.2(17)
C3	11.9(18)	18(2)	19(2)	2.8(18)	-0.1(18)	0.8(16)
C4	23(2)	19(2)	19(2)	-4.1(18)	-2(2)	0.1(19)
C5	27(2)	29(3)	13(2)	-0.7(19)	2(2)	1(2)
C6	21(2)	22(2)	20(2)	2.9(18)	5.1(19)	0.5(18)
C7	30(3)	25(2)	38(3)	8(2)	-9(3)	4(2)
C8	30(3)	24(2)	28(3)	-3(2)	1(2)	5(2)
N1	21.7(19)	20.3(19)	21(2)	-1.1(17)	4.0(17)	-0.3(16)
N2	22.0(19)	18.4(19)	21(2)	2.3(17)	0.2(18)	0.4(16)
O1	17.8(16)	27.4(18)	25.9(18)	-1.8(14)	0.0(15)	-7.3(13)
O2	31.0(18)	25.3(17)	20.1(17)	0.1(14)	-4.3(16)	-3.9(16)
O3	23.6(17)	26.2(17)	35(2)	4.6(16)	-9.1(17)	-8.7(14)
O4	23.3(17)	24.9(18)	32(2)	0.0(15)	11.3(16)	-0.5(14)
S1	17.4(5)	18.2(5)	18.7(6)	-1.1(4)	-0.8(4)	-2.8(4)
S2	16.5(5)	18.2(5)	21.2(5)	1.9(4)	0.8(4)	-2.5(4)
C9	16(2)	15(2)	21(2)	0.4(18)	-3.2(19)	0.7(17)
C10	23(2)	20(2)	22(3)	-5.3(18)	-6(2)	2.1(19)
C11	29(2)	27(2)	13(2)	-0.8(19)	-3(2)	3(2)
C12	21(2)	21(2)	19(2)	2.9(18)	-1.3(19)	-0.1(18)
C13	14.6(19)	18(2)	15(2)	-0.4(18)	-2.8(17)	1.3(16)
C14	18(2)	19(2)	16(3)	0.0(17)	0.6(18)	0.3(18)
C15	36(3)	24(2)	36(3)	-1(2)	4(3)	5(2)
C16	30(3)	25(2)	35(3)	3(2)	4(2)	5(2)
N3	20.0(19)	19.9(19)	18(2)	3.1(16)	-2.2(17)	1.4(15)
N4	24(2)	19.0(19)	23(2)	-0.9(17)	7.9(18)	-1.2(17)
O5	24.5(17)	23.2(18)	36(2)	0.9(16)	11.1(17)	-3.3(14)
O6	28.8(19)	25.2(18)	36(2)	3.6(16)	-13.0(18)	-9.1(15)
O7	28.9(18)	24.7(17)	20.7(17)	-0.2(14)	-3.7(16)	-3.8(15)
O8	20.4(16)	29.8(18)	23.8(18)	-1.5(14)	3.1(15)	-8.6(14)
S3	16.4(5)	16.8(5)	25.1(6)	1.4(5)	-1.0(4)	-2.3(4)
S4	17.8(5)	18.5(5)	15.9(5)	-1.2(4)	0.7(4)	-3.8(4)

Table S15: Bond Lengths in Å for **12**.

Atom	Atom	Length/Å
C1	C2	1.389(6)
C1	C6	1.383(7)
C1	S1	1.782(5)
C2	C3	1.393(7)
C3	C4	1.394(7)
C3	S2	1.774(5)
C4	C5	1.389(7)
C5	C6	1.391(7)
C7	N1	1.458(7)
C8	N2	1.462(7)
N1	S1	1.611(4)
N2	S2	1.614(4)
O1	S1	1.436(3)
O2	S1	1.436(4)
O3	S2	1.431(4)
O4	S2	1.442(4)

Atom	Atom	Length/Å
C9	C10	1.389(7)
C9	C14	1.383(7)
C9	S3	1.774(5)
C10	C11	1.389(7)
C11	C12	1.392(7)
C12	C13	1.388(7)
C13	C14	1.382(7)
C13	S4	1.764(5)
C15	N3	1.471(7)
C16	N4	1.474(7)
N3	S3	1.608(4)
N4	S4	1.614(4)
O5	S3	1.441(4)
O6	S3	1.433(4)
O7	S4	1.431(4)
O8	S4	1.435(3)

Table S16: Bond Angles in ° for **12**.

Atom	Atom	Atom	Angle/°
C2	C1	S1	117.0(4)
C6	C1	C2	122.0(5)
C6	C1	S1	121.0(4)
C1	C2	C3	117.8(5)
C2	C3	C4	121.8(5)
C2	C3	S2	117.8(4)
C4	C3	S2	120.3(4)
C5	C4	C3	118.5(5)
C4	C5	C6	121.1(5)
C1	C6	C5	118.9(5)
C7	N1	S1	120.5(4)
C8	N2	S2	118.8(4)
N1	S1	C1	107.6(2)
O1	S1	C1	107.5(2)
O1	S1	N1	107.6(2)
O1	S1	O2	119.3(2)
O2	S1	C1	107.5(2)
O2	S1	N1	106.9(2)
N2	S2	C3	108.5(2)
O3	S2	C3	107.7(2)
O3	S2	N2	107.6(2)
O3	S2	O4	119.0(2)
O4	S2	C3	107.4(2)
O4	S2	N2	106.2(2)

Atom	Atom	Atom	Angle/°
C10	C9	S3	120.4(4)
C14	C9	C10	120.8(5)
C14	C9	S3	118.8(4)
C9	C10	C11	119.6(5)
C10	C11	C12	120.0(5)
C13	C12	C11	119.7(5)
C12	C13	S4	121.2(4)
C14	C13	C12	120.7(5)
C14	C13	S4	118.1(4)
C13	C14	C9	119.4(5)
C15	N3	S3	118.9(4)
C16	N4	S4	118.6(4)
N3	S3	C9	108.8(2)
O5	S3	C9	107.1(2)
O5	S3	N3	105.7(2)
O6	S3	C9	106.9(2)
O6	S3	N3	108.0(2)
O6	S3	O5	119.9(2)
N4	S4	C13	107.5(2)
O7	S4	C13	107.7(2)
O7	S4	N4	106.6(2)
O7	S4	O8	118.8(2)
O8	S4	C13	108.2(2)
O8	S4	N4	107.5(2)

Table S17: Hydrogen Fractional Atomic Coordinates (×10[4]) and Equivalent Isotropic Displacement Parameters (Å[2]×10[3]) for**12**. U_{eq} is defined as 1/3 of the trace of the orthogonalised U_{ij}.

**Atom**	**x**	**y**	**z**	U_{eq}
H2	7352.84	4949.57	3451.35	20
H4	6670.48	4067.53	833.61	25
H5	8059.44	4920.57	74.78	28
H6	9076.78	5788.28	985.04	25
H7A	6715.35	6709.02	1655.48	46
H7B	8203.74	7193.24	1868.16	46
H7C	6414.11	7349.32	2283.16	46
H8A	7576.97	2802	1920.35	41
H8B	8836.53	2584.18	2748.58	41
H8C	9099.09	3255.75	2192.36	41
H1	7040.42	6730.05	3554.78	25
H2A	7424.99	3206.78	3799.67	24
H10	3268.61	5877.52	6667.64	26
H11	1934.73	5011.39	7427.2	28
H12	837.7	4169.75	6489.96	24
H14	2450.01	5051.61	4045.34	21
H15A	811.1	7320.01	4679.85	48
H15B	2272.7	7293.6	5453.61	48
H15C	963.02	6718.01	5404.21	48
H16A	3468.75	2655.87	5206.67	45
H16B	1714.9	2842.18	5646.36	45
H16C	3242	3318.72	5791.18	45
H3	2491.31	6766.16	3685.6	23
H4A	2806.02	3254.7	3892.32	26

Citations

O.V. Dolomanov and L.J. Bourhis and R.J. Gildea and J.A.K. Howard and H. Puschmann, Olex2: A complete structure solution, refinement and analysis program, *J. Appl. Cryst.*, (2009), **42**, 339-341.

Sheldrick, G.M. (2015). Acta Cryst. A71, 3-8.

Sheldrick, G.M. (2015). Acta Cryst. C71, 3-8.

H.D. Flack. Acta Cryst. A39, 876, (1983)
