# Supplementary material for: Conformational Analysis of Novel Benzene-1,3-Disulfonamide-Based Cycloalkynes Through X-Ray Crystallography, DFT Calculations, and NMR Spectroscopy
Source: Molecules. 2026 Jul 14;31(14):2462. doi: 10.3390/molecules31142462 (PMC13414055; doi:10.3390/molecules31142462)
Supplement: Supplementary file 1 [file molecules-31-02462-s001.zip › X-ray crystallography data of compound 7.pdf]

X-ray crystallography data of compound 7

R_1=2.97%

Crystal Data and Experimental

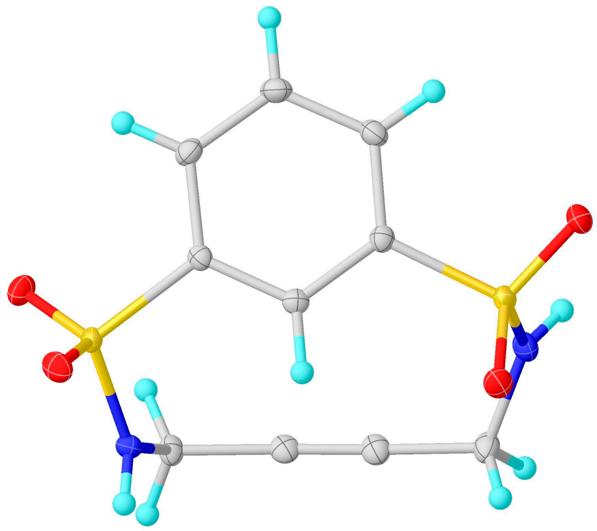

**Compound**	**7**
Formula	$C_{10}H_{10}N_2O_4S_2$
$D_{calc.}$/ g cm^{-3}	1.664
μ/mm^{-1}	4.343
Formula Weight	286.32
Colour	colourless
Shape	block
Size/mm^3	0.11×0.05×0.03
T/K	100.15
Crystal System	triclinic
Space Group	*P*-1
a/Å	6.6319(3)
b/Å	8.4791(3)
c/Å	11.2253(2)
α/°	75.219(3)
β/°	87.706(3)
γ/°	69.625(4)
V/Å^3	571.32(4)
Z	2
Z'	1
Wavelength/Å	1.54184
Radiation type	Cu K_α
Θ_{min}/°	4.079
Θ_{max}/°	77.768
Measured Refl.	24666
Independent Refl.	2427
Reflections with I > 2(I)	2349
R_{int}	0.0283
Parameters	164
Restraints	0
Largest Peak	0.639
Deepest Hole	-0.733
GooF	1.117
wR_2 (all data)	0.0834
wR_2	0.0831
R_1 (all data)	0.0303
R_1	0.0297

Experimental. Single colourless block-shaped crystals of **7** were obtained from slow evaporation of acetone at room temperature. A suitable crystal 0.11×0.05×0.03 mm^3 was selected and mounted on a MiTeGEN Dual Thickness MicroLoops in perfluoropolyether oil on an XtaLAB Synergy R, HyPix diffractometer. The crystal was kept at a steady T = 100.15 K during data collection. The structure was solved with the ShelXT (Sheldrick, 2015) structure solution program using the Intrinsic Phasing solution method and by using **Olex2** (Dolomanov et al., 2009) as the graphical interface. The model was refined with version 2018/3 of ShelXL 2018/3 (Sheldrick, 2015) using Least Squares minimisation.

Crystal Data. $C_{10}H_{10}N_2O_4S_2$, M_r = 286.32, triclinic, *P*-1 (No. 2), a = 6.6319(3) Å, b = 8.4791(3) Å, c = 11.2253(2) Å, α = 75.219(3)°, β = 87.706(3)°, γ = 69.625(4)°, V = 571.32(4) Å^3, T = 100.15 K, Z = 2, Z' = 1, μ(Cu K_α) = 4.343, 24666 reflections measured, 2427 unique (R_{int} = 0.0283) which were used in all calculations. The final wR_2 was 0.0834 (all data) and R_1 was 0.0297 (I > 2(I)).

ORTEP drawing of **7** showing thermal ellipsoids at the 50% probability level.

Structure Quality Indicators

**Reflections:**	d min (CuKα) 2Θ=155.5° 0.79	I/σ(I) 83.7	Rint m=10.16 2.83%	Full 135.4° 99% to 155.5° 100
**Refinement:**	Shift 0.001	Max Peak 0.6	Min Peak -0.7	GooF 1.117

Experimental Extended. A colourless block-shaped crystal with dimensions 0.11×0.05×0.03 mm^3 was mounted on a MiTeGEN Dual Thickness MicroLoops in perfluoropolyether oil. Data were collected using an XtaLAB Synergy R, HyPix diffractometer operating at T = 100.15 K.

Data were measured using ω scans using Cu K_α radiation. The diffraction pattern was indexed and the total number of runs and images was based on the strategy calculation from the program CrysAlisPro (Rigaku, V1.171.42.84a, 2023) The maximum resolution that was achieved was Θ = 77.768° (0.79 Å).

The diffraction pattern was indexed The diffraction pattern was indexed and the total number of runs and images was based on the strategy calculation from the program CrysAlisPro (Rigaku, V1.171.42.84a, 2023) and the unit cell was refined using CrysAlisPro (Rigaku, V1.171.42.84a, 2023) on 18543 reflections, 75% of the observed reflections.

Data reduction, scaling and absorption corrections were performed using CrysAlisPro (Rigaku, V1.171.42.84a, 2023). The final completeness is 100.00 % out to 77.768° in Θ. A multi-scan absorption correction was performed using CrysAlisPro 1.171.43.121a (Rigaku Oxford Diffraction, 2024) using spherical harmonics,implemented in SCALE3 ABSPACK scaling algorithm. The absorption coefficient μ of this material is 4.343 mm^{-1} at this wavelength (λ = 1.542Å) and the minimum and maximum transmissions are 0.817 and 1.291.

The structure was solved and the space group *P*-1 (# 2) determined by the ShelXT (Sheldrick, 2015) structure solution program using Intrinsic Phasing and refined by Least Squares using version 2018/3 of ShelXL 2018/3 (Sheldrick, 2015). All non-hydrogen atoms were refined anisotropically. Hydrogen atom positions were calculated geometrically and refined using the riding model. Hydrogen atom positions were calculated geometrically and refined using the riding model.

_exptl_absorpt_process_details: CrysAlisPro 1.171.43.121a (Rigaku Oxford Diffraction, 2024) using spherical harmonics,implemented in SCALE3 ABSPACK scaling algorithm.

Table S1: Fractional Atomic Coordinates (×10^4) and Equivalent Isotropic Displacement Parameters (Å^2×10^3) for **7.** U_{eq} is defined as 1/3 of the trace of the orthogonalised U_{ij}.

Atom	x	y	z	U_{eq}
C1	6674(2)	3756(2)	2673.8(14)	12.6(3)
C2	5527(2)	5470(2)	2692.4(14)	12.1(3)
C3	5312(2)	6769.5(19)	1610.4(14)	11.7(3)
S4	3533.0(6)	8912.1(4)	1555.3(3)	11.47(12)
N5	1547(2)	8759.8(17)	2393.6(12)	12.7(3)
C6	65(2)	7945(2)	2090.4(15)	14.6(3)
C7	665(2)	6111(2)	2799.0(15)	14.7(3)
C8	1303(3)	4664(2)	3447.3(15)	16.4(3)
C9	2267(3)	2898(2)	4268.8(16)	18.4(3)
N10	4292(2)	1824.0(17)	3859.1(13)	15.7(3)
S11	6564.4(6)	2080.5(5)	3967.2(3)	12.50(12)
C12	6340(3)	6398(2)	552.2(14)	14.9(3)
C13	7580(3)	4686(2)	584.7(15)	17.0(3)
C14	7716(3)	3345(2)	1632.6(15)	15.2(3)
O15	6558.3(19)	2708.4(15)	5044.6(10)	17.6(3)
O16	8211.0(19)	506.2(14)	3858.8(11)	17.7(3)
O17	2734.9(19)	9693.8(15)	298.1(10)	17.6(3)
O18	4605.7(19)	9742.2(15)	2152.0(11)	17.1(3)

Table S2: Anisotropic Displacement Parameters (×10[4]) **7.** The anisotropic displacement factor exponent takes the form: $-2\pi^2[h^2a^{*2} \times U_{11} + ... + 2hka^* \times b^* \times U_{12}]$

Atom	U_{11}	U_{22}	U_{33}	U_{23}	U_{13}	U_{12}
C1	11.9(7)	12.1(7)	13.0(7)	-2.1(6)	-0.1(5)	-4.1(6)
C2	10.8(7)	13.4(7)	12.4(7)	-3.6(6)	0.1(5)	-4.1(6)
C3	11.3(7)	11.3(7)	13.4(7)	-3.5(6)	0.8(5)	-4.8(6)
S4	11.82(19)	9.38(19)	11.8(2)	-1.12(14)	1.13(13)	-3.20(14)
N5	12.7(6)	12.3(6)	13.1(6)	-4.2(5)	2.6(5)	-3.8(5)
C6	12.4(7)	12.1(7)	17.2(7)	-1.0(6)	-0.9(6)	-3.6(6)
C7	11.7(7)	15.7(8)	17.6(7)	-4.5(6)	1.2(6)	-5.7(6)
C8	13.7(7)	15.5(8)	20.6(8)	-4.1(6)	1.7(6)	-6.3(6)
C9	15.6(8)	14.3(8)	21.5(8)	0.6(6)	4.3(6)	-4.6(6)
N10	17.0(7)	10.9(6)	18.8(7)	-4.0(5)	2.2(5)	-4.5(5)
S11	13.8(2)	9.87(19)	11.27(19)	-1.58(14)	0.25(13)	-1.82(14)
C12	17.5(8)	15.3(7)	12.9(7)	-2.4(6)	2.9(6)	-8.0(6)
C13	18.7(8)	18.0(8)	16.3(8)	-7.3(6)	6.3(6)	-7.4(6)
C14	14.4(7)	13.1(7)	18.3(8)	-6.1(6)	3.1(6)	-3.7(6)
O15	22.6(6)	16.0(6)	11.6(5)	-3.3(4)	-1.2(4)	-3.8(5)
O16	18.4(6)	11.8(5)	17.3(6)	-1.9(4)	1.8(4)	0.0(5)
O17	17.6(6)	17.3(6)	12.3(5)	1.7(4)	0.0(4)	-3.2(5)
O18	17.3(5)	14.7(5)	22.1(6)	-7.5(5)	2.8(5)	-7.2(4)

Table S3: Bond Lengths in Å for **7**.

Atom	Atom	Length/Å
C1	C2	1.391(2)
C1	S11	1.7729(15)
C1	C14	1.393(2)
C2	C3	1.390(2)
C3	S4	1.7754(16)
C3	C12	1.397(2)
S4	N5	1.6107(13)
S4	O17	1.4316(11)
S4	O18	1.4414(12)
N5	C6	1.476(2)

Atom	Atom	Length/Å
C6	C7	1.475(2)
C7	C8	1.192(2)
C8	C9	1.476(2)
C9	N10	1.468(2)
N10	S11	1.6095(14)
S11	O15	1.4397(12)
S11	O16	1.4305(12)
C12	C13	1.389(2)
C13	C14	1.393(2)

Table S4: Bond Angles in ° for **7**.

Atom	Atom	Atom	Angle/°
C2	C1	S11	117.88(12)
C2	C1	C14	121.54(14)
C14	C1	S11	120.31(12)
C3	C2	C1	118.18(14)
C2	C3	S4	118.58(12)
C2	C3	C12	121.45(14)
C12	C3	S4	119.82(12)
N5	S4	C3	107.87(7)
O17	S4	C3	106.27(7)
O17	S4	N5	108.62(7)
O17	S4	O18	119.48(7)
O18	S4	C3	108.81(7)
O18	S4	N5	105.34(7)
C6	N5	S4	121.75(11)

Atom	Atom	Atom	Angle/°
C7	C6	N5	111.78(13)
C8	C7	C6	172.75(17)
C7	C8	C9	175.04(17)
N10	C9	C8	114.39(14)
C9	N10	S11	123.91(11)
N10	S11	C1	107.74(7)
O15	S11	C1	106.93(7)
O15	S11	N10	107.38(7)
O16	S11	C1	107.16(7)
O16	S11	N10	106.99(7)
O16	S11	O15	120.12(7)
C13	C12	C3	118.92(15)
C12	C13	C14	120.76(15)
C13	C14	C1	118.91(14)

Table S5: Torsion Angles in ° for **7**.

Atom	Atom	Atom	Atom	Angle/°
C1	C2	C3	S4	170.93(11)
C1	C2	C3	C12	-4.5(2)
C2	C1	S11	N10	77.78(13)
C2	C1	S11	O15	-37.38(14)
C2	C1	S11	O16	-167.39(12)
C2	C1	C14	C13	-0.7(2)
C2	C3	S4	N5	-33.41(14)
C2	C3	S4	O17	-149.74(12)
C2	C3	S4	O18	80.39(13)
C2	C3	C12	C13	0.8(2)
C3	S4	N5	C6	-62.97(13)
C3	C12	C13	C14	3.1(2)
S4	C3	C12	C13	-174.57(12)
S4	N5	C6	C7	97.07(14)
C8	C9	N10	S11	74.80(18)
C9	N10	S11	C1	-83.32(14)
C9	N10	S11	O15	31.54(15)
C9	N10	S11	O16	161.74(12)
S11	C1	C2	C3	-169.65(11)
S11	C1	C14	C13	173.28(12)
C12	C3	S4	N5	142.12(13)
C12	C3	S4	O17	25.78(14)
C12	C3	S4	O18	-104.09(13)
C12	C13	C14	C1	-3.2(2)
C14	C1	C2	C3	4.5(2)
C14	C1	S11	N10	-96.41(14)
C14	C1	S11	O15	148.43(13)
C14	C1	S11	O16	18.42(15)
O17	S4	N5	C6	51.82(13)
O18	S4	N5	C6	-179.05(11)

Table S6: Hydrogen Fractional Atomic Coordinates (×10^4) and Equivalent Isotropic Displacement Parameters (Å^2×10^3) for **7**. U_{eq} is defined as 1/3 of the trace of the orthogonalised U_{ij}.

Atom	x	y	z	U_{eq}
H2	4906.66	5743.77	3424.57	15
H5	1339.85	9173.14	3049.75	15
H6A	-1419.79	8601.78	2270.9	18
H6B	83.8	8008.1	1197.35	18
H9A	2537.55	2999.58	5102.22	22
H9B	1214.54	2294.09	4337.28	22
H10	4260.26	990.29	3535.1	19
H12	6194.12	7302.2	-177.81	18
H13	8343.64	4427.19	-116.05	20
H14	8506.24	2169.55	1637.38	18

Citations

O.V. Dolomanov and L.J. Bourhis and R.J. Gildea and J.A.K. Howard and H. Puschmann, Olex2: A complete structure solution, refinement and analysis program, *J. Appl. Cryst.*, (2009), **42**, 339-341.

Sheldrick, G.M. (2015). Acta Cryst. A71, 3-8.

Sheldrick, G.M. (2015). Acta Cryst. C71, 3-8.
