# Supplementary material for: Conformational Analysis of Novel Benzene-1,3-Disulfonamide-Based Cycloalkynes Through X-Ray Crystallography, DFT Calculations, and NMR Spectroscopy
Source: Molecules. 2026 Jul 14;31(14):2462. doi: 10.3390/molecules31142462 (PMC13414055; doi:10.3390/molecules31142462)
Supplement: Supplementary file 1 [file molecules-31-02462-s001.zip › X-ray crystallography data of compound 8.pdf]

X-ray crystallography data of compound 8

R_1=3.27%

Crystal Data and Experimental

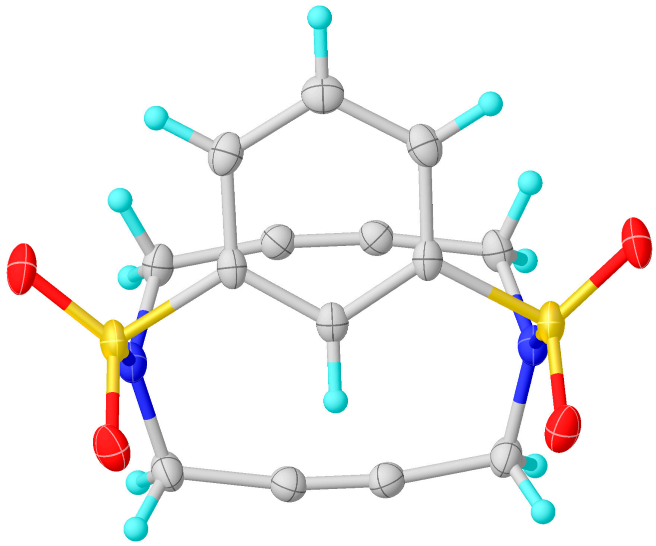

Experimental. Single colourless block-shaped crystals of **8** were obtained from slow evaporation of 1,4-dioxane at room temperature. A suitable crystal 0.06×0.05×0.03 mm^3 was selected and mounted on a MiTeGEN Dual Thickness MicroLoops in perfluoropolyether oil on an XtaLAB Synergy R, HyPix diffractometer. The crystal was kept at a steady T = 100.15 K during data collection. The structure was solved with the ShelXT (Sheldrick, 2015) structure solution program using the Intrinsic Phasing solution method and by using **Olex2** (Dolomanov et al., 2009) as the graphical interface. The model was refined with version 2018/3 of ShelXL 2018/3 (Sheldrick, 2015) using Least Squares minimisation.

Crystal Data. $C_{14}H_{12}N_2O_4S_2$, M_r = 336.38, orthorhombic, *Pnma* (No. 62), a = 11.8798(2) Å, b = 15.6230(3) Å, c = 7.46060(10) Å, $\alpha = \beta = \gamma = 90°$, V = 1384.67(4) Å^3, T = 100.15 K, Z = 4, Z' = 0.5, μ(Cu K$_\alpha$) = 3.689, 27997 reflections measured, 1532 unique (R_{int} = 0.0518) which were used in all calculations. The final wR_2 was 0.0953 (all data) and R_1 was 0.0327 (I > 2(I)).

ORTEP drawing of **8** showing thermal ellipsoids at the 50% probability level.

**Compound**	**8**
Formula	$C_{14}H_{12}N_2O_4S_2$
$D_{calc.}$/ g cm^{-3}	1.614
μ/mm^{-1}	3.689
Formula Weight	336.38
Colour	colourless
Shape	block
Size/mm^3	0.06×0.05×0.03
T/K	100.15
Crystal System	orthorhombic
Space Group	*Pnma*
a/Å	11.8798(2)
b/Å	15.6230(3)
c/Å	7.46060(10)
α/°	90
β/°	90
γ/°	90
V/Å^3	1384.67(4)
Z	4
Z'	0.5
Wavelength/Å	1.54184
Radiation type	Cu K$_\alpha$
Θ_{min}/°	5.664
Θ_{max}/°	77.727
Measured Refl.	27997
Independent Refl.	1532
Reflections with I > 2(I)	1447
R_{int}	0.0518
Parameters	103
Restraints	0
Largest Peak	0.434
Deepest Hole	-0.377
GooF	1.060
wR_2 (all data)	0.0953
wR_2	0.0939
R_1 (all data)	0.0342
R_1	0.0327

Structure Quality Indicators

**Reflections:**	d min (CuKα) 2Θ=156.1° 0.79	I/σ(I) 54.3	Rint m=19.65 5.18%	Full 135.4° 99% to 156.1° 100
**Refinement:**	Shift 0.001	Max Peak 0.4	Min Peak -0.4	GooF 1.060

Experimental Extended. A colourless block-shaped crystal with dimensions 0.06×0.05×0.03 mm^3 was mounted on a MiTeGEN Dual Thickness MicroLoops in perfluoropolyether oil. Data were collected using an XtaLAB Synergy R, HyPix diffractometer operating at T = 100.15 K.

Data were measured using ω scans using Cu K_α radiation. The diffraction pattern was indexed and the total number of runs and images was based on the strategy calculation from the program CrysAlisPro (Rigaku, V1.171.42.84a, 2023) The maximum resolution that was achieved was Θ = 77.727° (0.79 Å).

The diffraction pattern was indexed The diffraction pattern was indexed and the total number of runs and images was based on the strategy calculation from the program CrysAlisPro (Rigaku, V1.171.42.84a, 2023) and the unit cell was refined using CrysAlisPro (Rigaku, V1.171.42.84a, 2023) on 13936 reflections, 50% of the observed reflections.

Data reduction, scaling and absorption corrections were performed using CrysAlisPro (Rigaku, V1.171.42.84a, 2023). The final completeness is 100.00 % out to 77.727° in Θ. A multi-scan absorption correction was performed using CrysAlisPro 1.171.43.121a (Rigaku Oxford Diffraction, 2024) using spherical harmonics,implemented in SCALE3 ABSPACK scaling algorithm. The absorption coefficient μ of this material is 3.689 mm^{-1} at this wavelength (λ = 1.542Å) and the minimum and maximum transmissions are 0.845 and 1.000.

The structure was solved and the space group *Pnma* (# 62) determined by the ShelXT (Sheldrick, 2015) structure solution program using Intrinsic Phasing and refined by Least Squares using version 2018/3 of ShelXL 2018/3 (Sheldrick, 2015). All non-hydrogen atoms were refined anisotropically. Hydrogen atom positions were calculated geometrically and refined using the riding model. Hydrogen atom positions were calculated geometrically and refined using the riding model.

_exptl_absorpt_process_details: CrysAlisPro 1.171.43.121a (Rigaku Oxford Diffraction, 2024) using spherical harmonics,implemented in SCALE3 ABSPACK scaling algorithm.

Table S7: Fractional Atomic Coordinates (×10^4) and Equivalent Isotropic Displacement Parameters (Å^2×10^3) for **8.** U_{eq} is defined as 1/3 of the trace of the orthogonalised U_{ij}.

Atom	x	y	z	U_{eq}
C1	3311.7(14)	3814.8(9)	3817(2)	24.2(3)
C2	3165.1(12)	2882.3(10)	3637(2)	22.4(3)
N3	4053.8(10)	4061.4(8)	5321.4(17)	21.2(3)
S4	3527.5(3)	4198.2(2)	7337.5(5)	21.24(16)
C5	3891.7(12)	3260.0(9)	8528.4(19)	20.0(3)
C6	4811.8(13)	3271.5(9)	9692(2)	23.3(3)
C7	5235.0(18)	2500	10327(3)	24.7(4)
C8	3388.3(17)	2500	7988(3)	19.9(4)
O9	4124.5(10)	4889.8(7)	8167.7(15)	28.0(3)
O10	2331.4(10)	4253.2(7)	7125.7(17)	27.9(3)
C11	5256.9(12)	3816.8(9)	5201(2)	22.4(3)
C12	5442.0(11)	2881.8(10)	5287.7(19)	19.9(3)

Table S8: Anisotropic Displacement Parameters (×10⁴) **8.** The anisotropic displacement factor exponent takes the form: $-2\pi^2[h^2a^{*2} \times U_{11} + ... +2hka^* \times b^* \times U_{12}]$

Atom	U_{11}	U_{22}	U_{33}	U_{23}	U_{13}	U_{12}
C1	32.0(7)	17.0(7)	23.6(7)	1.6(5)	-3.1(6)	3.5(6)
C2	23.2(7)	22.0(6)	22.0(7)	-0.1(6)	-2.7(6)	1.5(5)
N3	27.1(7)	15.1(5)	21.5(6)	0.1(5)	0.9(5)	0.4(4)
S4	28.3(3)	12.2(2)	23.2(2)	-0.85(12)	4.19(13)	1.21(11)
C5	25.6(7)	14.3(7)	20.1(6)	-0.3(5)	4.9(5)	1.0(5)
C6	28.8(7)	20.5(7)	20.4(7)	-3.0(5)	4.1(6)	-3.3(5)
C7	26.6(10)	28.7(11)	18.8(9)	0	0.1(8)	0
C8	22.3(9)	16.3(9)	20.9(9)	0	4.8(7)	0
O9	43.3(6)	14.1(5)	26.6(6)	-3.3(4)	3.6(5)	-3.1(4)
O10	28.2(6)	21.3(6)	34.2(6)	1.9(4)	5.8(5)	6.4(4)
C11	25.7(7)	16.6(7)	24.8(7)	0.2(5)	4.0(5)	-3.2(5)
C12	19.1(6)	20.4(6)	20.2(6)	0.3(5)	1.0(5)	-1.0(5)

Table S9: Bond Lengths in Å for **8**.

Atom	Atom	Length/Å
C1	C2	1.473(2)
C1	N3	1.4782(19)
C2	C2[1]	1.195(3)
N3	S4	1.6428(13)
N3	C11	1.4822(18)
S4	C5	1.7677(15)
S4	O9	1.4332(11)
S4	O10	1.4323(13)
C5	C6	1.396(2)
C5	C8	1.3893(18)
C6	C7	1.3891(18)
C11	C12	1.479(2)
C12	C12[1]	1.193(3)

----
[1]+x,1/2-y,+z

Table S10: Bond Angles in ° for **8**.

Atom	Atom	Atom	Angle/°
C2	C1	N3	113.43(12)
C2[1]	C2	C1	171.41(8)
C1	N3	S4	120.13(10)
C1	N3	C11	117.51(12)
C11	N3	S4	117.13(10)
N3	S4	C5	105.02(7)
O9	S4	N3	107.79(7)
O9	S4	C5	106.66(7)
O10	S4	N3	106.52(7)
O10	S4	C5	110.37(7)
O10	S4	O9	119.56(7)
C6	C5	S4	119.58(11)
C8	C5	S4	117.22(12)
C8	C5	C6	121.90(14)
C7	C6	C5	118.96(14)
C6[1]	C7	C6	120.38(19)
C5	C8	C5[1]	117.5(2)
C12	C11	N3	113.29(12)
C12[1]	C12	C11	171.08(8)

----
[1]+x,1/2-y,+z

Table S11: Torsion Angles in ° for **8**.

Atom	Atom	Atom	Atom	Angle/°
C1	N3	S4	C5	102.54(11)
C1	N3	S4	O9	-144.02(11)
C1	N3	S4	O10	-14.55(13)
C1	N3	C11	C12	-67.01(17)
C2	C1	N3	S4	-87.67(15)
C2	C1	N3	C11	66.07(17)
N3	S4	C5	C6	99.95(12)
N3	S4	C5	C8	-67.29(14)
S4	N3	C11	C12	87.53(14)
S4	C5	C6	C7	-166.62(13)
S4	C5	C8	C5[1]	161.64(10)
C5	C6	C7	C6[1]	5.5(3)

Atom	Atom	Atom	Atom	Angle/°
C6	C5	C8	C5[1]	-5.3(3)
C8	C5	C6	C7	0.0(2)
O9	S4	C5	C6	-14.28(14)
O9	S4	C5	C8	178.48(13)
O10	S4	C5	C6	-145.62(12)
O10	S4	C5	C8	47.14(15)
C11	N3	S4	C5	-51.30(12)
C11	N3	S4	O9	62.15(12)
C11	N3	S4	O10	-168.39(10)

––––

[1]+x,1/2-y,+z

Table S12: Hydrogen Fractional Atomic Coordinates (×10^4) and Equivalent Isotropic Displacement Parameters (Å^2×10^3) for **8**. U_{eq} is defined as 1/3 of the trace of the orthogonalised U_{ij}.

Atom	x	y	z	U_{eq}
H1A	2564.44	4082.33	3993.91	29
H1B	3630.66	4043.04	2688.37	29
H6	5143.27	3798.63	10044.45	28
H7	5817.2	2499.99	11199.45	30
H8	2725.16	2500.01	7276.57	24
H11A	5569.13	4036.35	4059.8	27
H11B	5673.45	4093.46	6193.53	27

Citations

O.V. Dolomanov and L.J. Bourhis and R.J. Gildea and J.A.K. Howard and H. Puschmann, Olex2: A complete structure solution, refinement and analysis program, *J. Appl. Cryst.*, (2009), **42**, 339-341.

Sheldrick, G.M. (2015). Acta Cryst. A71, 3-8.

Sheldrick, G.M. (2015). Acta Cryst. C71, 3-8.
